# Impact of Pore Flexibility in Imine-Linked Covalent Organic Frameworks on Benzene and Cyclohexane Adsorption

**DOI:** 10.1021/acsami.2c09911

**Published:** 2022-08-30

**Authors:** Marco Moroni, Esther Roldan-Molina, Rebecca Vismara, Simona Galli, Jorge A. R. Navarro

**Affiliations:** † Dipartimento di Scienza e Alta Tecnologia, Università dell’Insubria, Via Valleggio 11, 22100 Como, Italy; ‡ Departamento de Química Inorgánica, Universidad de Granada, Avenida de Fuentenueva S/N, 18071 Granada, Spain; § Instituto de Investigaciones Químicas, CSIC-Universidad de Sevilla, Calle Américo Vespucio 49, 41092 Seville, Spain; ∥ Consorzio Interuniversitario Nazionale per la Scienza e Tecnologia dei Materiali, 50121 Firenze, Italy

**Keywords:** covalent organic frameworks, COF-300, LZU-111, liquid organic hydrogen carriers, gas separation

## Abstract

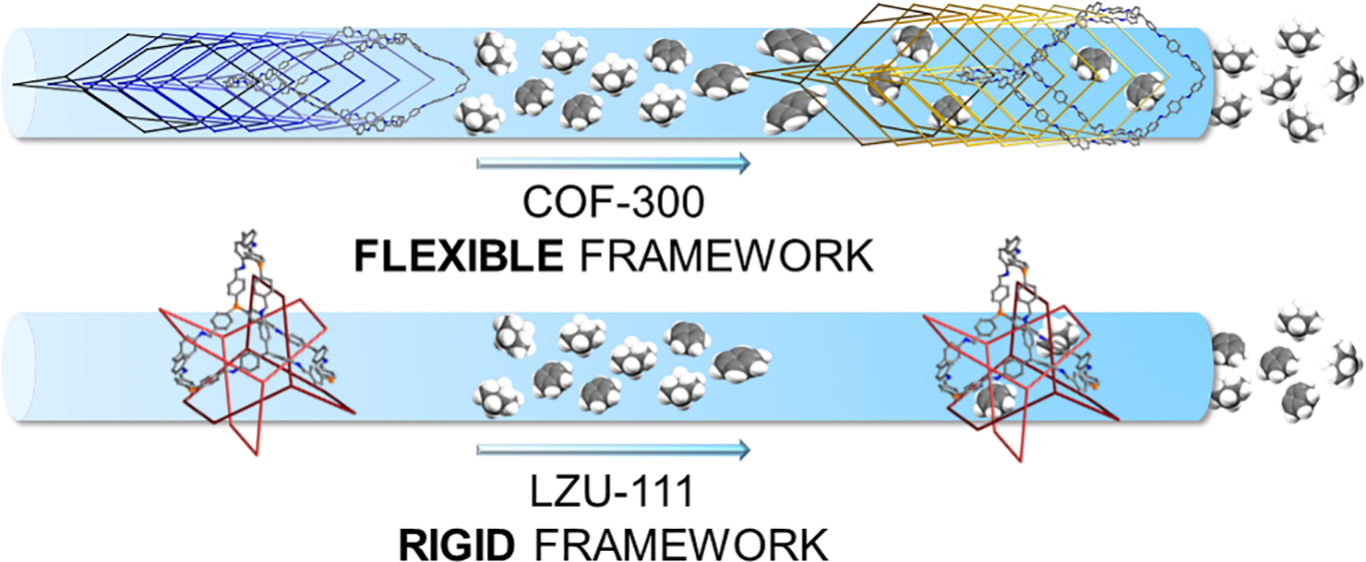

This work focuses on the impact of covalent organic frameworks’ (COFs) pore flexibility in the adsorption and separation of benzene and cyclohexane. With this aim, we have selected the imine-linked 3D COFs COF-300 and LZU-111 as examples of flexible and rigid frameworks, respectively. Optimized syntheses at room temperature or in solvothermal conditions enabled us to selectively isolate the narrow-pore form of COF-300 (COF-300-rt) or a mixture of the narrow-pore and a larger-pore form (COF-300-st), respectively, with different textural properties (BET specific surface area = 39 or 1270 m^2^/g, respectively, from N_2_ adsorption at 77 K). In the case of LZU-111, only the room temperature route was successful, leading to the known microporous framework. COF-300-rt, COF-300-st, and LZU-111 were studied for benzene and cyclohexane adsorption and separation in static and dynamic conditions. At 298 K and 1 bar, these COFs adsorb more benzene (251, 221, and 214 cm^3^/g STP, respectively) than cyclohexane (175, 133, and 164 cm^3^/g STP, respectively). Moreover, the benzene and cyclohexane isotherms of COF-300-rt and COF-300-st are characterized by steps, as expected with a flexible material. Indeed, *in situ* powder X-ray diffraction experiments on benzene- and cyclohexane-impregnated batches enabled us to trap, for the first time, a sequence of forms of COF-300 with different pore aperture, rationalizing the stepped hysteretic isotherms. Finally, benzene/cyclohexane separation was evaluated using a benzene/cyclohexane 50:50 v/v flow at different temperatures (*T* = 298, 323, and 348 K): LZU-111 does not selectively retain any of the two components, while COF-300 exhibits stronger benzene–COF interactions also in dynamic conditions.

## Introduction

Among the 17 Sustainable Development Goals set by the United Nations in 2015,^1^ Goal 7, *Affordable and clean energy*, triggers the access to sustainable and renewable energy forms. In this context, the so-called hydrogen economy^2^ has decisively returned to the scene.

Hydrogen can be considered as a future energy carrier for stationary and mobile applications.^3^ Nonetheless, a smooth transition to the hydrogen economy is possible only after overcoming some unsolved challenges, including the construction and diffusion of large-scale infrastructures, or the cost of hydrogen production, storage and transportation. Hydrogen can undergo physical-based storage as a compressed gas or as a liquid.^4^ However, storage as gas requires high-pressure tanks (sustaining 350–700 bar) and storage as liquid requires cryogenic temperatures [*T*_b H_2__ = 20.3 K]. Both approaches are costly and pose safety issues for on-board applications. A more effective alternative is material-based storage, based on physisorption in porous solids, or on chemisorption by hydrides and organic liquids.^4^

Liquid organic hydrogen carriers (LOHCs)^5^ are organic compounds that can reversibly and selectively absorb and release hydrogen by means of hydrogenation and dehydrogenation catalytic reactions. As such, LOHCs can be used as versatile hydrogen storage media. The optimal LOHCs are liquid at ambient conditions and possess properties similar to those of crude oil-based liquids. Hence, they can be handled, transported, and stored, taking advantage of the existing crude oil-based infrastructures.^6^ Moreover, LOHCs show high gravimetric and volumetric hydrogen density and release high purity (close to 100%) hydrogen.

One example of LOHCs could be the cyclohexane (CH)/benzene (BEN) couple.^7^ After hydrogenation, unreacted benzene is inevitably present in the reactor effluent stream and must be removed to recover pure cyclohexane. Sequestration of benzene from the mixture is complicated and energy consuming.^8,9^ As a matter of fact, cyclohexane and benzene show comparable boiling points (*T*_b CH_ = 351 K, *T*_b BEN_ = 350 K) and form an azeotrope, with this occurrence leading to inefficient fractional distillation. Azeotropic distillation^10^ and extractive distillation,^11^ the latter performed in the presence of entrainers, can separate the two liquids but require high operating costs and complex processes to achieve the aimed high purity. An alternative approach is the selective physisorption of cyclohexane or benzene on porous solids, taking advantage of their different kinetic diameters (Ø_CH_ = 6.00 Å, Ø_BEN_ = 5.85 Å)^12^ or of the preferential interactions that one of the two can establish with the adsorbent pore walls. This approach not only could reduce the energy footprint but also it does not produce waste.^13^

Covalent organic frameworks (COFs)^14^ are open frameworks built through the condensation of organic monomers *via* covalent bonds. Since the seminal work of Yaghi and co-workers in 2005,^15^ COFs modular construction, enabled by a variety of building blocks, has led to periodically homogeneous porosity in which pore dimension and pore wall functionality can be tuned. This has opened the way to different potential applications, ranging from separation^16^ to heterogeneous catalysis,^17^ environmental remediation,^18^ and optoelectronic processes.^19^ Imine-based COFs show a number of advantages with respect to other COFs:^20^ for example, they can be obtained in a variety of experimental conditions, including room temperature, and they show a higher chemical stability than other COFs.

**Scheme 1:**
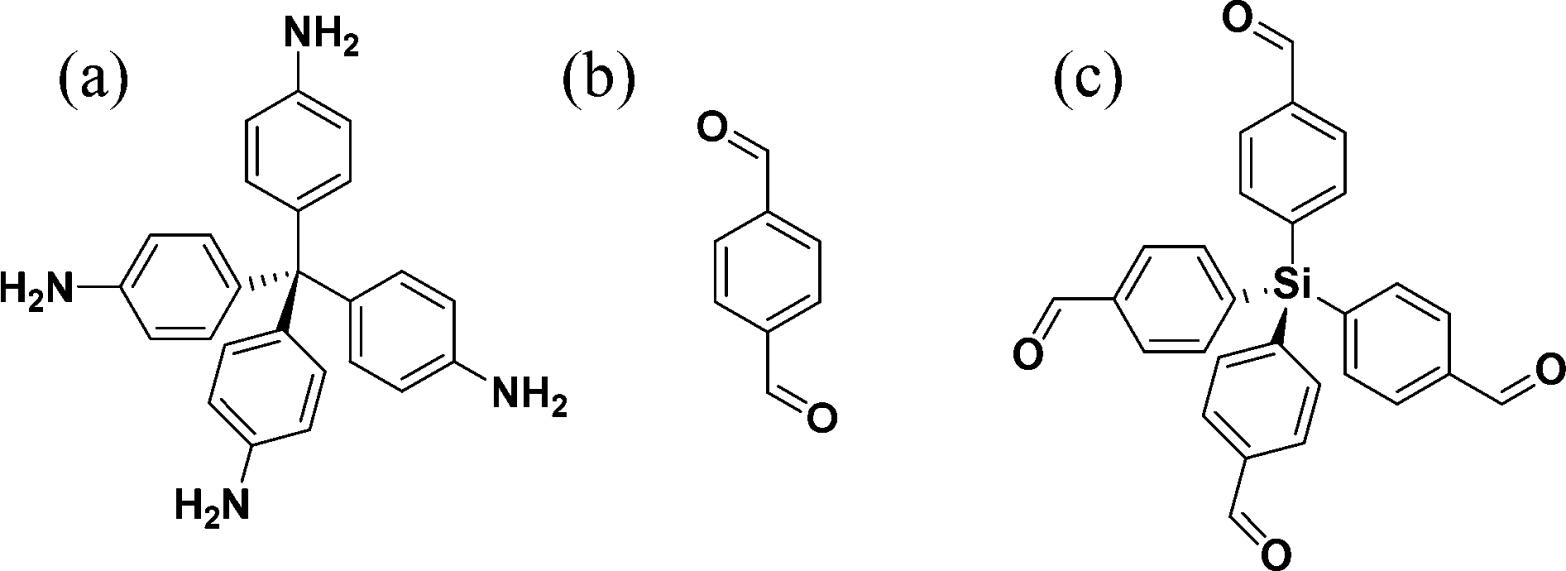
Molecular Structure of (a) Tetrakis(4-aminophenyl)methane, TAM, (b) Terephthaldehyde, BDA, and (c) Tetrakis(4-formylphenyl)silane, TFS

**Figure 1:**
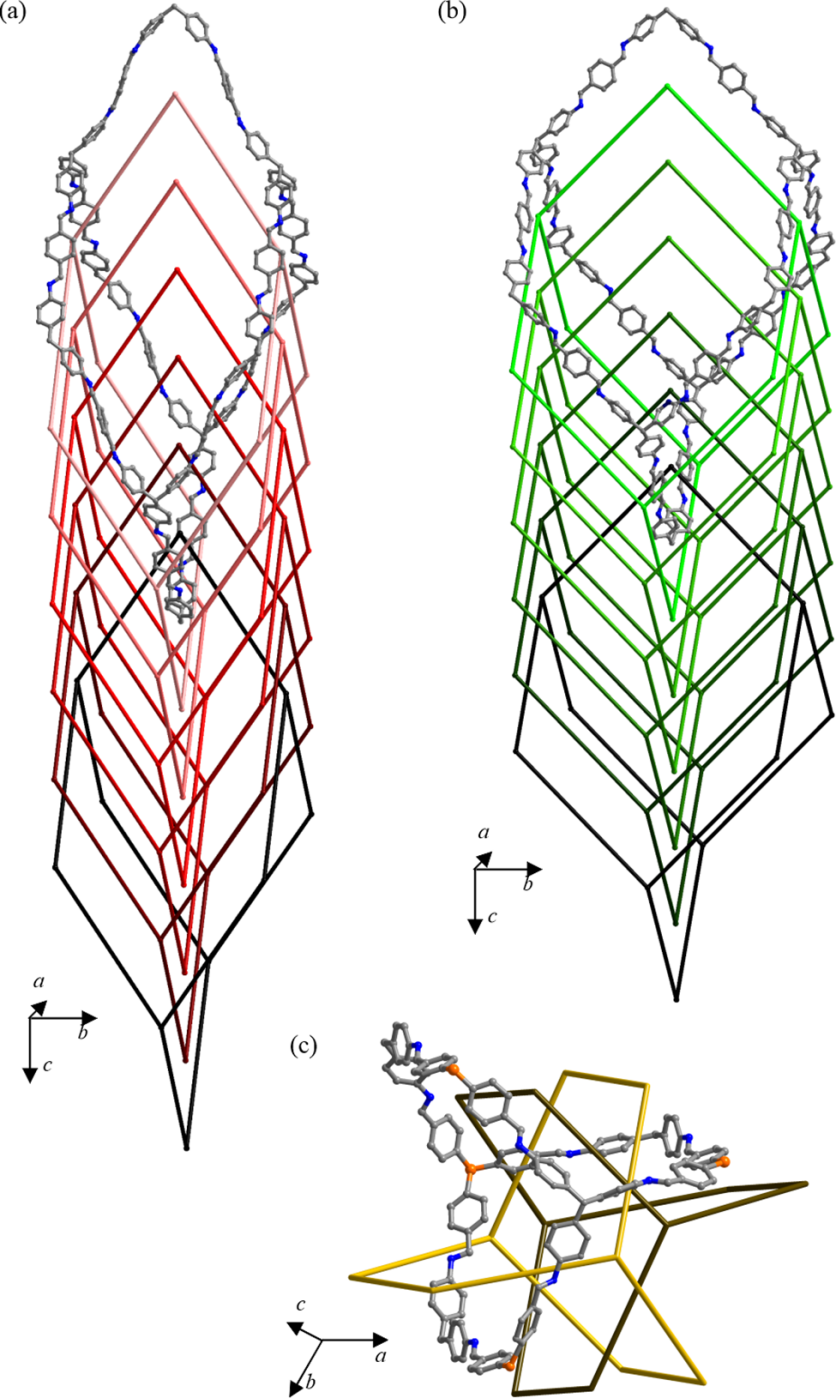
Portion of one of the interpenetrated frameworks in (a) the narrow-pore form of COF-300 (crystallographic information from 24), (b) the large-pore forms of COF-300 (crystallographic information from 24) and (c) LZU-111 (crystallographic information from 22). The 7-fold interpenetration of COF-300 and the 3-fold interpenetration of LZU-111 are schematically highlighted. Atom color code: carbon, gray; nitrogen, blue; silicon, orange. Hydrogen atoms have been omitted for clarity.

The imine-based covalent organic framework [(TAM)(BDA)] [COF-300; TAM = tetrakis(4-aminophenyl)methane, BDA = terephthaldehyde, Scheme 1a,b], first appeared in 2009,^21^ crystallizes in a tetragonal space group, and shows a 7-fold interpenetrated framework of diamondoid topology, with tetrahedral nodes and 1D linear channels decorated by the aromatic rings of the TAM and BDA monomers (Figure 1a,b). COF-300 is known to be *flexible*: two different forms have been isolated so far as a function of synthesis conditions or external stimuli, the narrow-pore form (NP, Figure 1a; also named hydrated,^22^ collapsed,^23^ or COF-300-H_2_O^24^ in the literature) and the large-pore one (LP, Figure 1b).^22^

On the other hand, the imine-based covalent organic framework [(TAM)(TFS)] [LZU-111; TFS = tetrakis(4-formylphenyl)silane, Scheme 1c]^22^ crystallizes in a hexagonal space group and shows a 3-fold interpenetrated *rigid* framework having lonsdaleite topology, with tetrahedral nodes and 1D helicoidal channels (Figure 1c). Also in this case, the channels are decorated by the aromatic moieties of the monomers. Given the specificity of their pore walls decoration, COF-300 and LZU-111 seem ideal candidates to be exploited in the separation of benzene and cyclohexane: the former should be selectively retained due to the formation of preferential host–guest π–π interactions. While, in the case of metal–organic frameworks, several works have been published on the separation of the two vapors,^25^ in the case of COFs, few articles exist on their static adsorption,^26−29^ and only one study has been released, to the best of our knowledge, on their dynamic adsorption and separation.^30^

In the following, we report on the results of the benzene and cyclohexane adsorption studies carried out on COF-300 and LZU-111 in static and dynamic conditions, demonstrating the crucial role of structural flexibility, with the formation of intermediate-pore and large-pore forms, in the adsorption and separation of the two vapors.

## Experimental Section

### Materials and Methods

Commercial chemicals were acquired from vendors and used as received, without further purification. Tetrakis(4-aminophenyl)methane (TAM) was synthesized according to the procedure reported in Section S1 of the Supporting Information (SI). Figures S1–S6 show the ^1^H and ^13^C NMR spectra of the intermediates and of TAM. The purity and crystallinity degree of any sample of the COFs used for the structural and functional characterization were assessed by powder X-ray diffraction (PXRD). The PXRD patterns were acquired with a Bruker AXS θ:θ geometry diffractometer equipped with an X-ray sealed source (Cu Kα, λ = 1.5418 Å), a filter of nickel in the diffracted beam, a Bruker Lynxeye linear position sensitive detector, and the following optics: Soller slits in the incident and diffracted beams (2.5° aperture), divergence slit (0.5° aperture), and receiving slit (8 mm height). The generator was set at 40 kV and 40 mA. Scanning electron microscopy images were acquired with a Philips XL30 ESEM-FEG scanning electron microscope, working at 20 kV and 170 μA and with a spot size of 3.0, and employing a secondary electrons detector. The samples (ca. 5 mg) were deposited on a disk of smooth carbon tape; then, they were covered with a 10 μm layer of gold. The images were acquired with a sample to detector distance of 15 mm. ^1^H and ^13^C NMR spectra were recorded on a Bruker Avance Neo instrument (500 MHz) using CDCl_3_ or DMSO-*d*_6_ as solvents. The chemical shifts (δ) are expressed in parts per million and the coupling constants (*J*) in Hertz. The multiplicity is indicated with the following abbreviations: br (broad), s (singlet), d (doublet), t (triplet), q (quartet), and m (multiplet). The Fourier transform infrared (FTIR) spectra of COF-300-rt, COF-300-st, and LZU-111 were recorded on a Bruker Tensor 27 spectrometer. Powdered samples (1 mg) were mixed with anhydrous KBr (100 mg). The mixture was pressed at 5 tons into a 12 mm diameter pellet using a Specac hydraulic press. A pure KBr pellet was used as a blank. The FTIR spectra were collected in the 4000–400 cm^–1^ range at a resolution of 4 cm^–1^. The band maximum positions are given in wavenumbers (cm^–1^). The bands are described as very strong (vs), strong (s), medium (m), weak (w), very weak (vw), or broad (br).

### Room-Temperature Synthesis of COF-300 (COF-300-rt)

In a Pyrex bottle, at room temperature and under sonication, BDA (120 mg, 0.89 mmol) and aniline (0.6 mL, 6.58 mmol) were dissolved in dioxane (5 mL). After complete dissolution, 6 M aqueous acetic acid (4 mL) was added to the solution under sonication. In another Pyrex bottle, TAM (200 mg, 0.52 mmol) was dissolved in dioxane (5 mL) under sonication, and the obtained solution was added to the first one. The resulting mixture was sonicated for a few seconds, until a homogeneous mixture was formed. Then, the bottle was closed with a cap, and the mixture was allowed to stand at room temperature for 3 days. A yellow precipitate was formed. The suspension was then centrifuged (15 min, 3700 rpm). The mother liquor was removed, anhydrous tetrahydrofuran (THF) (50 mL) was added, and the suspension was centrifuged again (15 min, 3700 rpm). Then, the liquid was removed and the solid was immersed in new THF (50 mL) for 24 h at room temperature to exchange with dioxane. Finally, the mother liquor was removed, and the precipitate was preliminarily dried with a flow of air; then, it was put in an oven at 383 K for 2 h. Yield: 222 mg (82% based on TAM). IR (KBr, cm^–1^): 3377 (br), 3060 (vw), 3028 (w), 2960 (vw), 2924 (vw), 2884 (w), 2851 (vw), 1919 (w), 1697 (s), 1618 (vs), 1589 (s), 1509 (s), 1494 (vs), 1415 (m), 1366 (w), 1301 (m), 1199 (m), 1174 (m), 1113 (w), 1014 (w), 973 (m), 916 (w), 878 (m), 841 (s), 800 (m), 745 (w), 718 (w), 554 (m), and 413 (m) (Figure S7).

### Solvothermal Synthesis of COF-300 (COF-300-st)

In a Pyrex bottle, at room temperature and under sonication, BDA (60 mg, 0.45 mmol) and TAM (100 mg, 0.26 mmol) were dissolved in dioxane (5 mL). After complete dissolution, 6 M aqueous acetic acid (1 mL) was added under sonication, and the bottle was closed with a septum. Then, the reaction mixture was cooled in liquid N_2_ under a vacuum (10^–3^ bar) for 5 min. After room temperature was reached again, the bottle was sealed with a cap (over the septum) and put in an oven at 393 K for 3 days. A yellow precipitate was formed, which was vacuum filtered and washed with dioxane (10 mL) and THF (10 mL). Then, the solid was immersed in THF (50 mL) for 24 h at room temperature. Finally, the mother liquor was removed, and the precipitate was preliminarily dried with a flow of air; then, it was put in an oven at 373 K for 4 h. Yield: 86 mg (64% based on TAM). IR (KBr, cm^–1^): 3378 (br), 3060 (vw), 3028 (w), 2959 (vw), 2924 (vw), 2884 (w), 2851 (vw), 1919 (w), 1697 (s), 1618 (vs), 1589 (s), 1509 (s), 1494 (vs), 1415 (m), 1366 (w), 1301 (m), 1199 (m), 1174 (m), 1113 (w), 1014 (w), 974 (m), 916 (w), 878 (m), 841 (s), 800 (m), 745 (w), 718 (w), 554 (m), and 413 (m) (Figure S7).

### Synthesis of LZU-111

In a Pyrex bottle, at room temperature and under sonication, TFS (89.6 mg, 0.20 mmol) and aniline (0.72 mL, 7.90 mmol) were dissolved in dioxane (2 mL). After complete dissolution, 6 M aqueous acetic acid (1.6 mL) was added to the solution under sonication. In another Pyrex bottle, TAM (76 mg, 0.20 mmol) was dissolved in dioxane (2 mL) under sonication, and the obtained solution was added to the first one. The resulting mixture was sonicated for a few seconds, after sealing the bottle with a cap; then, it was left still at room temperature for 3 days. An off-white precipitate was formed. The suspension was then transferred into a centrifuge tube and centrifuged (15 min, 3700 rpm). The mother liquor was removed, anhydrous THF (50 mL) was added, and the suspension was centrifuged again (15 min, 3700 rpm). Then, the liquid was removed, and the resulting solid was immersed in new THF (50 mL) for 24 h at room temperature. Finally, the suspension was vacuum filtered, and the off-white powder was put in an oven at 383 K for 2 h. Yield: 94 mg (57% based on TAM). All the attempts carried out to isolate LZU-111 through a solvothermal synthesis, varying the synthesis conditions, resulted in an unknown low crystallinity solid (PXRD evidence), which was not investigated further. IR (KBr, cm^–1^): 3375 (br), 3066 (w), 3026 (m), 2957 (w), 2868 (w), 2734 (w), 2300 (w), 1929 (w), 1703 (s), 1624 (vs), 1672 (w), 1597 (m), 1551 (m), 1509 (m), 1494 (m), 1394 (w), 1381 (m), 1362 (w), 1311 (w), 1267 (w), 1208 (m), 1174 (m), 1016 (m), 976 (w), 886 (w), 820 (s), 762 (w), 697 (vs), 638 (w), 585 (m), 508 (w), and 413 (m) cm^–1^. (Figure S7).

### Variable-Temperature Powder X-ray Diffraction

The variable-temperature powder X-ray diffraction experiments were carried out acquiring the data in isothermal conditions from 298 to 758 K (the highest temperature that can be reached by the device) with steps of 20 K, using a custom-made sample heater (Officine Elettrotecniche di Tenno, Ponte Arche, Italy) plugged in the Bruker AXS diffractometer described above. The acquisitions were carried out in the 2θ range collected in Table 1 with a step of 0.02° and a time per step of 1 s.

**Table 1:** Conditions Used for the VT-PXRD Experiments Performed on COF-300-rt, COF-300-st, and LZU-111

COF	*T*, K	2θ, °
COF-300-rt	298–758	7.0–25.5
	298–478–298	7.0–25.5
COF-300-st	298–758	4.5–23.0
LZU-111	298–758	4.0–19.5

Samples of COF-300-rt, COF-300-st, and LZU-111 (ca. 30 mg) were deposited in the hollow of an aluminum sample-holder 0.1 mm deep. The data acquired before a significant loss of crystallinity were treated by means of a whole powder pattern refinement with the Le Bail method^31^ using the program TOPAS-R v.3.^32^ As a starting point, we adopted the unit cell parameters reported in the literature.^22^ The background was described through a Chebyshev polynomial function. The peak profile was modeled with the so-called Fundamental Parameters Approach.^33^ For COF-300-rt and LZU-111, the anisotropic shape of the peaks was modeled with spherical harmonics of proper order. In the case of COF-300-rt, a second experiment was performed, heating the COF from 298 K up to 478 K and then decreasing the temperature down to 298 K. The PXRD data were acquired every 20 K, with a step of 0.02° and a time per step of 1 s. The data were then treated as described above.

### Textural Properties Assessment

The N_2_ adsorption isotherms were measured at 77 K under continuous adsorption conditions using a Micromeritics 3Flex adsorption analyzer, taking advantage of a liquid N_2_ bath with 99.999% purity. Prior to the measurement, the samples (ca. 70–90 mg) were activated at 393 K under high vacuum (10^–6^ Torr) for 12 h. To identify the pressure range of the N_2_ isotherm where to apply the Brunauer–Emmett–Teller (BET) model to estimate the specific surface area, we adopted the consistency criteria described by Rouquerol and co-workers.^34^ Further details about the estimation of the BET specific surface area are provided in Section S4 of the SI.

### Benzene and Cyclohexane Adsorption in Static Conditions

The benzene and cyclohexane adsorption isotherms were measured at 298 K under continuous adsorption conditions with the Micromeritics 3Flex adsorption analyzer quoted in the previous section. Prior to the measurement, the samples (ca. 70–90 mg) were activated at 393 K under a high vacuum (10^–6^ Torr) for 12 h. High-purity (>99%) benzene and cyclohexane were used as sources of the vapors. To ensure the absence of any dissolved gas, two freeze–thaw cycles were applied to the two solvents prior to the measurement.

### Benzene and Cyclohexane Adsorption in Dynamic Conditions

The breakthrough curves of benzene, cyclohexane, and a 50:50 v/v mixture of benzene/cyclohexane were collected at three different temperatures (298, 323, and 348 K) taking advantage of the oven of a gas chromatograph. Mixtures of He/benzene, He/cyclohexane, or He/benzene/cyclohexane were flowed (20 mL/min) in a stainless-steel column (length = 8, 7, and 15 cm for COF-300-rt, COF-300-st, and LZU-111, respectively; internal diameter = 0.6 cm) packed with powdered samples (ca. 500 mg) of COF-300-rt, COF-300-st, or LZU-111. The relative gas mixture composition exiting the column (He, benzene, and cyclohexane molar masses = 4.00, 78.06, and 84.12 a.m.u., respectively) was monitored by employing a quadrupole Omnistar mass spectrometer; helium was used as a reference to estimate the dead volume of the column. Prior to the measurement, the samples were activated at 393 K under a flow of He (20 mL/min) for 12 h. Reactivation was performed after each measurement before performing another one, with heating under a flow of He (20 mL/min) at 453 K for 40 min and at 463 K for 20 min.

### Following Benzene and Cyclohexane Adsorption and Desorption by *in Situ* Powder X-ray Diffraction

The structural behavior of COF-300-rt, COF-300-st, and LZU-111 triggered by impregnation with benzene and cyclohexane was monitored by PXRD using the diffractometer described above. In a typical experiment, after deposing a weighted quantity (ca. 20 mg) of powdered sample on an aluminum sample-holder 0.4 mm deep, a PXRD pattern was acquired in the 2θ range reported in Table 2 with steps of 0.02° and a time per step of 1 s. Then, leaving the sample in the sample-holder, an aliquot (100 μL) of benzene or cyclohexane was dropped using a micropipette to completely impregnate the sample. A number of consecutive PXRD acquisitions were performed on the impregnated material with the same experimental conditions of the preliminary PXRD measurement, until no changes in the diffraction pattern were observed compared with the preceding one. Since as-synthesized COF-300-st and LZU-111 contain open-pore forms, as assessed by PXRD and N_2_ adsorption (*vide infra*), prior to each experiment they were activated under a vacuum (10^–3^ Torr) at 393 K for 12 h. A first impregnation experiment was carried out in a wider 2θ range (Table 2). The PXRD data of COF-300-rt and LZU-111 were treated carrying out a whole powder pattern refinement performed with the Le Bail method^31^ using TOPAS-R v.3.^32^

**Table 2:** Conditions Adopted for the PXRD Experiments Performed on COF-300-rt, COF-300-st, and LZU-111 before and after Impregnation with Benzene or Cyclohexane

COF	2θ, °
COF-300-rt	5.0–25.5
	5.5–13.0
COF-300-st	5.0–25.5
LZU-111	4.0–19.5

The background was described through a Chebyshev polynomial function. The peak profile was modeled with the so-called Fundamental Parameters Approach.^33^

The anisotropic shape of the peaks was modeled with spherical harmonics of proper order. In the case of COF-300-rt, immediately after the impregnation, a partial closure of the thus formed larger-pore form occurred during the acquisition. To account for this occurrence, these data were treated using two separate regions (in the 2θ ranges 5.8–16.0° and 16.0–25.0° for benzene and 5.5–16.2° and 16.2–25.5° for cyclohexane). In the case of COF-300-st, the low data quality did not allow for any treatment. To increase the monitoring frequency on COF-300-rt, another impregnation experiment was carried out in a smaller 2θ range (Table 2). Again, the data were modeled by means of a whole powder pattern refinement, as described above. Finally, in the case of COF-300-rt, as the narrow-pore forms recovered after both impregnations with benzene and cyclohexane showed slightly wider unit cell volumes than the pristine sample, the samples were left in the sample-holder and monitored at different time lapses, up to 42 days, until an asymptote was reached.

## Results and Discussion

### Synthesis

COF-300 was obtained in the form of microcrystalline powders (3–9 μm, see below) applying two distinct synthetic paths, derived from previously reported ones, namely, at room-temperature and under solvothermal conditions.

**Figure 2:**
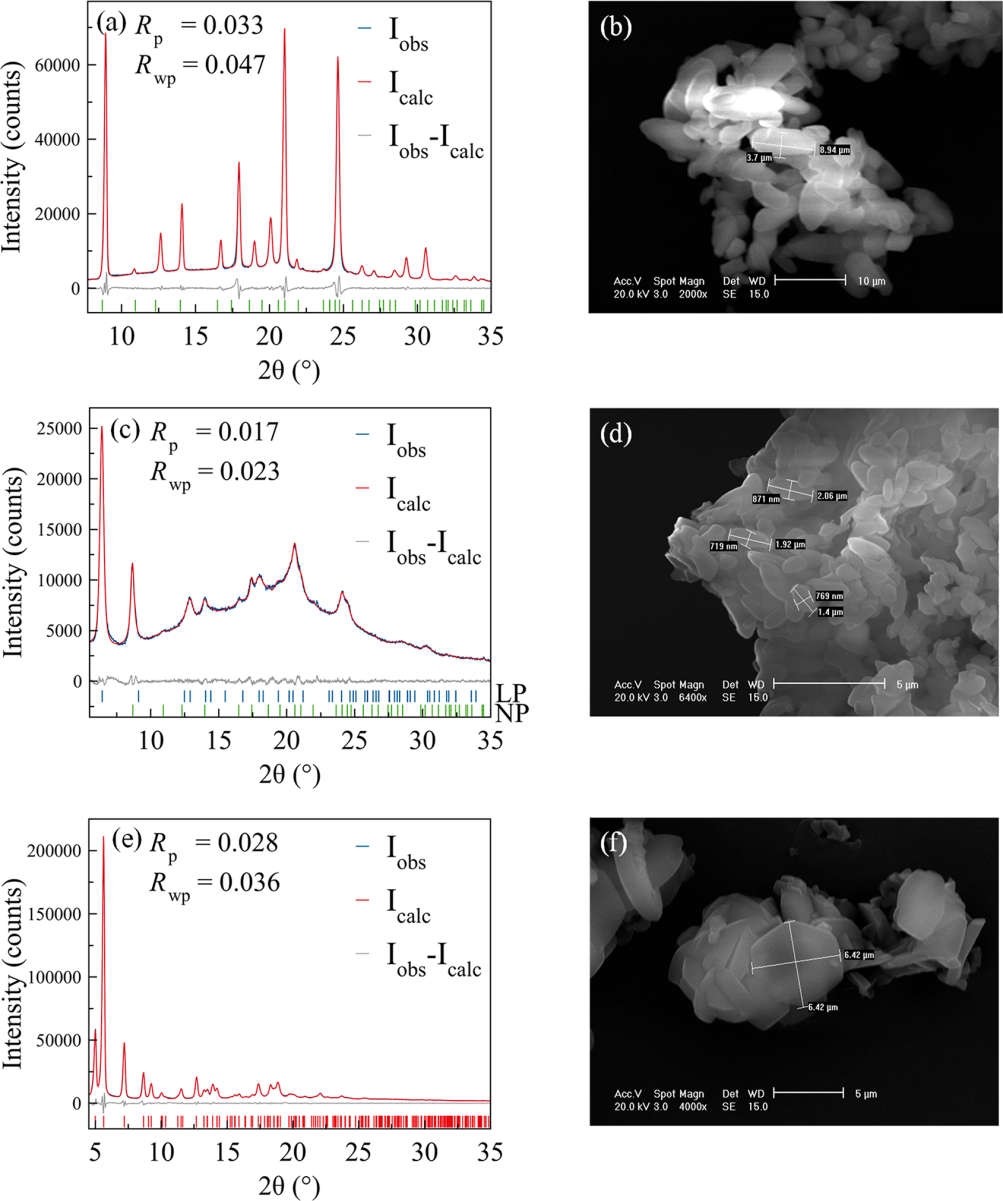
Whole powder pattern refinements and SEM images of (a and b) COF-300-rt, (c and d) COF-300-st, and (e and f) LZU-111. In a, c, e, observed, calculated, and different traces in blue, red, and gray, respectively. The ticks at the bottom indicate the positions of the Bragg reflection maxima.

As assessed by powder X-ray diffraction (Figure 2a), the room-temperature path yielded samples (COF-300-rt) only containing the narrow-pore form. As expected, the latter hosts water molecules in its channels (as proved by the broad IR spectroscopy band at 3377 cm^–1^, ascribed to O–H stretching; Figure S7). These samples show a higher crystallinity than those isolated by Fischbach and co-workers in 2019,^23^ but comparable to that reported by Chen and co-workers^24^ or Ma and co-workers.^35^ Fischbach et al. aged the reaction mixture at 363 K for 2 days, followed by 1 day at room temperature. Ma et al. proposed a more structured synthetic process requiring at least 7 days of aging at room and high temperature.^35^ In the present case, the synthesis proceeds along 3 days exclusively at room temperature and without degassing, which is a typical procedure for COFs synthesis, or ventilation.^24^ To improve crystallinity, we used aniline as a modulator, as already done for the growth of both single crystals^22^ and powders.^35^ Scanning electron microscopy (Figure 2b and Figure S8a,b for the images; Figure S9 for the statistics) indicated the isolation of crystallites of ca. 8–9 μm for the larger dimension.

On the other hand, the synthesis we carried out in solvothermal conditions, omitting a modulator, yielded a lower-crystallinity mixture (COF-300-st; Figure 2c for the powder X-ray diffraction pattern) of the narrow-pore form and a larger-pore form, as already occurred to Fischbach et al.^23^ The latter worked at 363 K for 2 days in biphasic conditions, followed by 1 day additional soaking, while we aged in monophasic conditions for 3 days at 393 K. Worthy of note, the powder X-ray diffraction pattern of COF-300-st resembles that reported in 2009^21^ and interpreted with the purported 5-fold interpenetrated form labeled dia-5. The crystal structure of dia-5 was not determined from the powder diffraction pattern, but it was proposed based on calculations, starting from a unit cell not perfectly describing all the powder pattern. The hypothesis of 5-fold interpenetration was never confirmed by a successive structure determination. Hence, we suggest that, also in 2009, a mixture of forms with different pore apertures, like in COF-300-st, was isolated. Scanning electron microscopy images of a COF-300-st sample (Figure 2d and Figure S8c,d for the images; Figure S9 for the statistics) revealed smaller crystallites than in the case of COF-300-rt, namely, of ca. 3–4 μm for the larger dimension, in line with the absence of a modulator, which lowers the crystallization rate.

Finally, LZU-111 was prepared in 57% yield with 3 days of aging at room temperature, isolating samples of the known form^22^ (Figure 2e for the powder X-ray diffraction pattern), containing hexagonal crystallites of ca. 6 μm dimension (Figure 2f and Figure S8e,f for the images; Figure S9 for the statistics). In the recent past, LZU-111 powders of similar size (1 μm) have been prepared following a longer 7-day aging procedure at room temperature and high temperature, preceded by freezing in N_2_.^35^ As proven by IR spectroscopy (O–H stretching witnessed by the broad band at 3375 cm^–1^; Figure S7), LZU-111 hosts water molecules in its channels. The attempts carried out to isolate LZU-111 through a solvothermal synthesis, varying the synthesis conditions, yielded an unknown low-crystallinity phase that was not investigated further.

### Thermal Behavior

**Figure 3:**
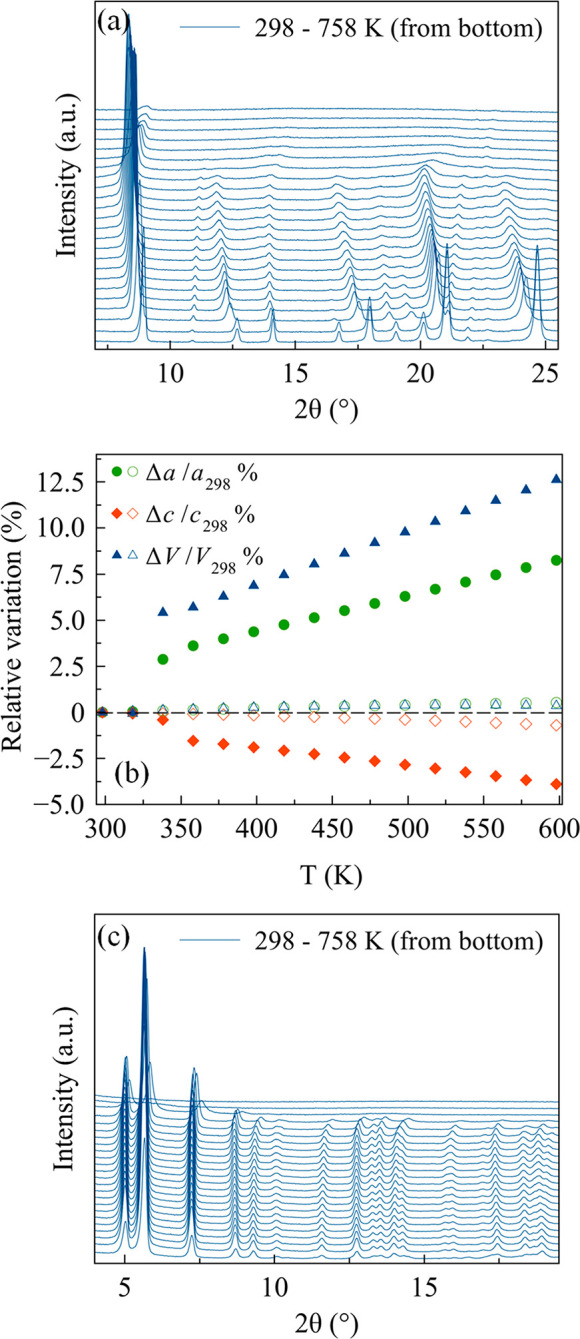
(a) Powder X-ray diffraction patterns of COF-300-rt acquired as a function of the temperature. From the bottom: 298 to 758 K with steps of 20 K. (b) Comparison between the percentage relative variation of the unit cell parameters (*a*, *c*, *V*) of COF-300-rt (full symbols) and LZU-111 (empty symbols). (c) Powder X-ray diffraction patterns of LZU-111 acquired as a function of the temperature. From the bottom: 298 to 758 K with steps of 20 K.

As described in the Experimental Section, two different VT-PXRD experiments were carried out in air on COF-300-rt. Figure 3a collects the powder X-ray diffraction patterns acquired, with steps of 20 K, in the temperature range 298–758 K.

As highlighted above, COF-300-rt only contains the narrow-pore form, with a unit cell volume comparable to those reported in the literature for the so-called hydrated COF-300^22,24^ (the value in ref (22) was retrieved at 100 K). COF-300-rt starts losing crystallinity at 618 K, and it is almost completely amorphous at 758 K.^36^ This observation agrees with some of the previously reported decomposition onset temperatures (773^23^ and 723 K^24^). More interestingly, upon increasing the temperature, a non-negligible shift to the left of the positions of the Bragg reflections occurs, suggesting an increase of the unit cell volume. The shift is particularly pronounced in the temperature range 318–338 K. As a matter of fact, a parametric whole powder pattern refinement (Figure S10a) revealed that the *a*-axis and, above all, the unit cell volume undergo a significant variation in this temperature range (Δ*a*/*a* = 2.9%, Δ*c*/*c* = −0.3%, Δ*V*/*V* = 5.5%; volumetric thermal expansion coefficient α_V_ = 2700 × 10^–6^ K^–1^; Figure 3b). The observed unit cell volume variation agrees with the loss of the water molecules hosted in the pores, as previously discussed in the literature.^24^ Indeed, in the present case, the increase of the *a*-axis implies an increase of the channels’ diameter, which is compatible with the breaking of the host–guest hydrogen bonds upon water release. As a complement to this, the variation of the [020] and [040] peaks intensity witnesses a change in the electronic density within the channels. Above 338 K, the unit cell parameters variation follows the same trend (the *a*-axis and the volume increase, the *c*-axis decreases) but with a lower steepness (358–598 K, Δ*a*/*a* = 4.6%, Δ*c*/*c* = −2.3%, Δ*V*/*V* = 6.9%, α_V_ = 270 × 10^–6^ K^–1^; 298–598 K, Δ*a*/*a* = 8.3%, Δ*c*/*c* = −3.9%, Δ*V*/*V* = 12.6%, α_V_ = 420 × 10^–6^ K^–1^).

The larger-pore form obtained in this case has a volume of 3900(4) Å^3^, which is lower than that reported in the literature for the largest-pore form observed, COF-300-THF^24^ [5503.7(5) Å^3^; value retrieved at 100 K], but it is comparable to that of the activated phase labeled COF-300-V.^24^ To the best of our knowledge, this is the first time in which a sequence of forms of COF-300 with different pore aperture are observed and characterized. Noteworthy, a 478 to 298 K cooling experiment (Figure S11a of the SI) suggests that the pore aperture triggered by the temperature increase is reversible. This is confirmed by the data treatment (Figure S11b): excluding a small hysteresis at about 338 K, the unit cell parameters values of the cooling branch are almost completely superimposed with those of the heating branch (Figure S11c).

As anticipated, COF-300-st contains a mixture of the narrow-pore form and a larger-pore form [V = 4928(3) Å^3^]. This occurrence holds true for all the investigated thermal range (298–758 K) (see Figure S12a of the SI). As disclosed with a whole powder pattern parametric refinement (Figure S12b), the narrow-pore form behaves as described above for COF-300-rt (Figure S12c), while the larger-pore form shows only a moderate variation of the unit cell parameters (maximum variation in the 298–598 K range: Δ*a*/*a* = 1.6%, Δ*c*/*c* = −1.0%, Δ*V*/*V* = 2.1%, α_V_ = 71 × 10^–6^ K^–1^; Figure S12d). Despite the slight difference in crystal size, there is no difference between the thermal behavior of COF-300-rt and the narrow pore form in COF-300-st, as previously observed for samples of different crystal size by means of thermogravimetric analysis.^35^

Finally, Figure 3c collects the powder X-ray diffraction patterns of LZU-111 acquired, as a function of the temperature, in the range 298–758 K. This COF undergoes a slight crystallinity increase on passing from 298 to 318 K, starts losing crystallinity at 678 K, and is almost completely amorphous at 758 K. As assessed by a whole powder pattern parametric data treatment (Figure S10b), the framework is rigid, showing a variation of the unit cell parameters lower than 1% (298–618 K: Δ*a*/*a* = 0.6%, Δ*c*/*c* = −0.8%, Δ*V*/*V* = 0.3%; α_V_ = 10 × 10^–6^ K^–1^; Figure 3b). Hence, an order of magnitude difference exists between the unit cell parameters’ thermal expansion of COF-300-rt and LZU-111. This striking difference in framework rigidity among the two systems can be appreciated in Figure 3b.

### Textural Properties

The permanent porosity of samples of COF-300-rt, COF-300-st, and LZU-111 was evaluated acquiring volumetric N_2_ adsorption isotherms at 77 K after thermal activation (see the Experimental Section).

Noteworthy, in the case of COF-300-rt and COF-300-st, thermal activation does leave the samples unaltered from the point of view of the pore dimension or the forms ratio (PXRD evidence, Figure S13).

**Figure 4:**
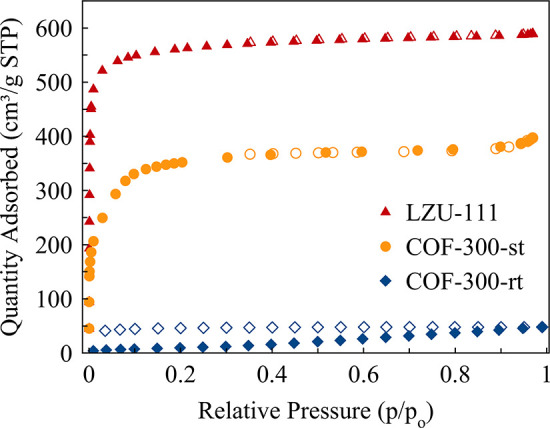
N_2_ adsorption isotherms acquired at 77 K on COF-300-rt, COF-300-st, and LZU-111. Empty symbols are descriptive of the desorption branch.

COF-300-rt and COF-300-st adsorb different amounts of N_2_ (Figure 4). COF-300-rt shows a type III adsorption isotherm and it is not porous to N_2_, possessing a Brunauer–Emmett–Teller (BET) specific surface area of only 39 m^2^/g, in accordance with what was reported in the literature for the so-called collapsed form (21 m^2^/g).^23^ The observed hysteresis loop is reasonably indicative of a guest-induced form change related to the flexible nature of the framework. The pore distribution was calculated by the 2D-NLDFT method.^37^ As expected, COF-300-rt shows limited porosity in the 35 Å region (see Figure S14 in the SI).

At variance, COF-300-st, showing a type I adsorption isotherm, displays a BET specific surface area of 1270 m^2^/g, in agreement with the value of 1360 m^2^/g reported in the literature^21^ for what is possibly a mixture of the narrow-pore form and a larger-pore form (*vide supra*), as in the present case. The pore distribution was calculated by the 2D-NLDFT method: two different pore types were found at 9.7 and 16.2 Å (Figure S14).

Worthy of note, no steps or hysteresis were observed along the N_2_ isotherm of COF-300-st, indicating that no gate opening from the narrow-pore form to a larger-pore form occurs during adsorption.

Differently from what was observed in the recent past,^35^ the different amounts of N_2_ adsorbed by COF-300-rt and COF-300-st cannot be related to the slight difference in crystal size. The two batches show different crystallinity (i.e., defectivity), as witnessed by the full width at half maximum of their diffraction peaks (Figure 2a,c). This could be responsible for the different N_2_ adsorption, as observed by Ma and co-workers.^35^ Nonetheless, COF-300-st contains a larger pore-form. As no evidence of flexibility was observed when dosing N_2_ on COF-300-st, we believe that the presence of the larger-pore form has a major role in allowing N_2_ adsorption.

Finally, LZU-111 displays a type I adsorption isotherm typical of a microporous rigid material (Figure 4), with a BET specific surface area of 1840 m^2^/g, confirming what is reported in the scientific literature.^22,35^ The pore distribution was calculated by the 2D-NLDFT method. LZU-111 shows pores of 10 Å (Figure S14), which are in good agreement with the values reported in the scientific literature.^35^

### Benzene and Cyclohexane Adsorption in Static Conditions

**Figure 5:**
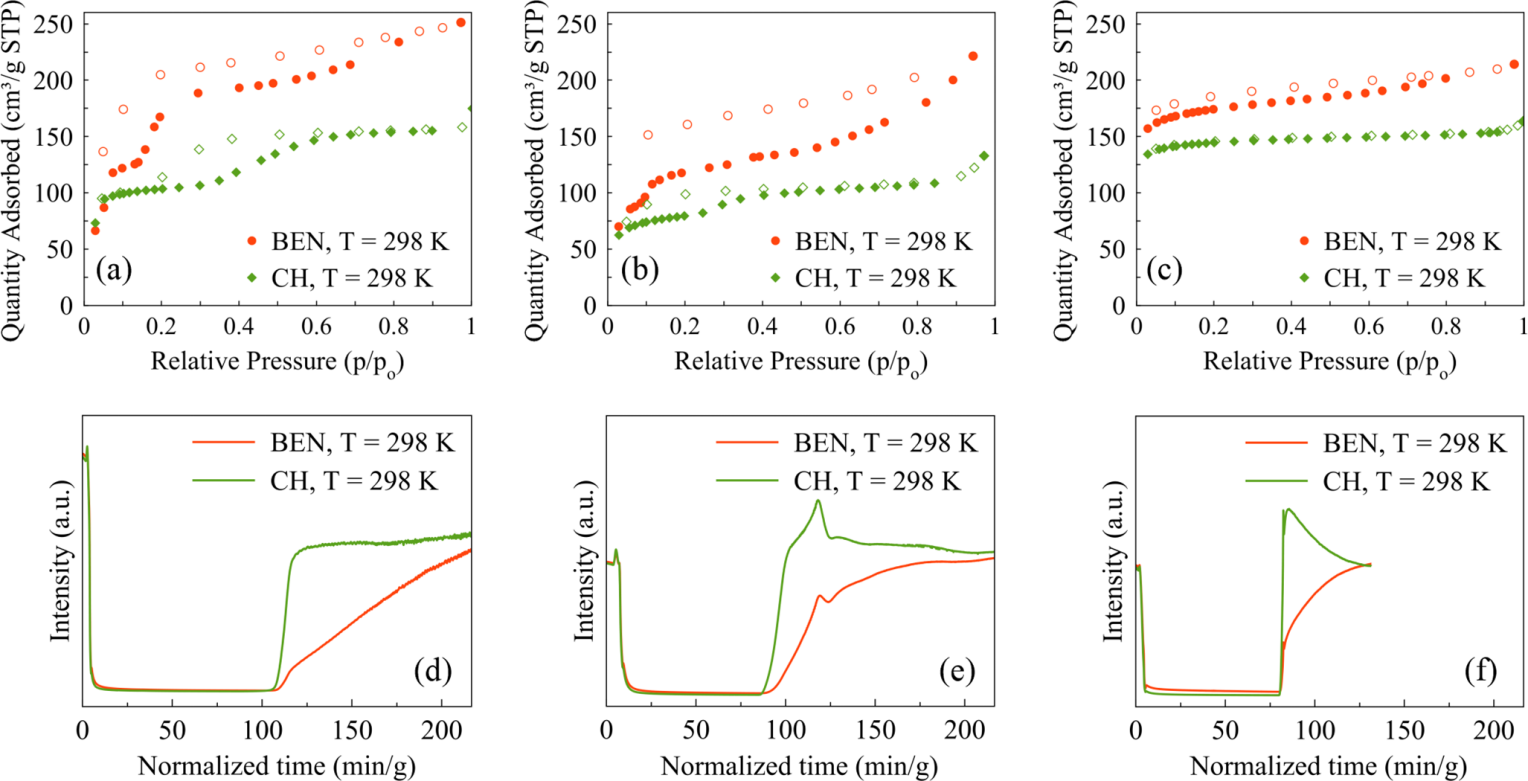
Benzene and cyclohexane adsorption isotherms acquired at 298 K on (a) COF-300-rt, (b) COF-300-st, and (c) LZU-111. Empty symbols describe the desorption branch. Breakthrough curves of (d) COF-300-rt, (e) COF-300-st, and (f) LZU-111 flowing a 50:50 v/v mixture of benzene and cyclohexane at 298 K. For the breakthrough curves of the 50:50 v/v mixture at 323 and 348 K, the reader is addressed to Figures S15 and S16 of the SI, while for those of the single gases at 298, 323, and 348 K to Figures S17–S19.

The benzene and cyclohexane adsorption isotherms measured on COF-300-rt are of type IV/VI (Figure 5a). In all the explored pressure range (*p*/*p*_0_ = 0–1), this sample adsorbs more benzene than cyclohexane, with 251 and 175 cm^3^/g STP (11.2 and 7.8 mmol/g) adsorbed, respectively, at *p*/*p*_0_ = 1. This occurrence nicely shows that the fact that the narrow-pore form is poorly accessible to N_2_ does not exclude applications relying on porosity, as it was recently claimed.^23^ For both vapors, the adsorption and desorption branches are characterized by two steps, indicating a progressive aperture/closure of the pores starting from/going back to the narrow-pore form, as observed by PXRD (see below). Along the adsorption branches, the two *plateaux* begin at *p*/*p*_0_ of ca. 0.07 and 0.29 for benzene and at *p*/*p*_0_ of ca. 0.05 and 0.59 for cyclohexane. The adsorption branch between the first and the second plateaux is steeper in the case of benzene, suggesting a higher affinity of the COF for this vapor. Both desorption branches show hysteresis. In the case of benzene, the desorption branch is never superimposed to the adsorption one, suggesting an uncomplete pore closure with respect to the pristine narrow-pore form. This occurrence can be tentatively ascribed to the formation of preferential (π–π) interactions among benzene and the pore walls, increasing the affinity for this vapor and making it more difficult to be released, thus stabilizing a form with intermediate pore aperture.

A similar behavior was observed for COF-300-st (Figure 5b), even though in this case the steps (i.e., the gate openings) are less definite, possibly because a larger-pore form is already present together with the narrow-pore one. For this sample, the maximum quantity adsorbed at *p*/*p*_0_ = 1 is 221 and 133 cm^3^/g STP (9.9 and 5.9 mmol/g) for benzene and cyclohexane, respectively.

At variance, in the case of LZU-111 the adsorption isotherms of benzene and cyclohexane (Figure 5c) are of type I, with a very small hysteresis along that of benzene, confirming the rigidity of this COF. At *p*/*p*_0_ = 1, LZU-111 adsorbs 214 and 164 cm^3^/g STP (9.6 and 7.3 mmol/g) of benzene and cyclohexane, respectively.

To the best of our knowledge, a few examples exist of benzene or cyclohexane adsorption by COFs (Table 3). As for benzene adsorption, COF-300-st, COF-300-rt, and LZU-111 perform better than TPPE-COF, similarly to COF-1 and CTF-IP-10, and worse than TBICOF. In the case of cyclohexane, they perform better than COF-1 and they are similar to the best performing TBICOF.

**Table 3:** Quantity of Benzene and Cyclohexane Adsorbed at 293/298 K and 1 bar by Selected COFs

COF	*T*, K	*p*, bar	Ads. BEN, cm^3^/g STP	Ads. CH, cm^3^/g STP	ref
COF-300-rt	298	1	251	175	a
COF-300-st	298	1	221	133	a
LZU-111	298	1	214	164	a
COF-1	298	1	220	87	(26)
CTF-IP-10	298	1	280	negligible	(27)
TBICOF	298	1	641.9	186.2	(28)
TTPE-COF	293	1	131.6	n.a.	(29)

a This work.

### Benzene and Cyclohexane Adsorption in Dynamic Conditions

As a complement to the benzene and cyclohexane adsorption studies in static conditions, we took advantage of breakthrough curves to investigate the adsorption of these volatile organic compounds also in dynamic conditions and to study the ability of COF-300-rt, COF-300-st, and LZU-111 in their capture and separation. Only one example exists of benzene/cyclohexane separation by COFs, showing at 333 K a slightly higher retention time for benzene.^30^

Parts d–f of Figure 5 gather the benzene and cyclohexane breakthrough curves for COF-300-rt, COF-300-st, and LZU-111, respectively, acquired for a 50:50 v/v mixture at 298 K, while Figures S15 and S16 collect the breakthrough curves at 323 and 348 K, respectively. At 298 K, the results are indicative of an initial coadsorption of the two vapors by all the materials. COF-300-rt (Figure 5d) displays a retention time of ca. 117 and 100 min/g for benzene and cyclohexane, respectively, after which the material starts to become saturated. Interestingly, the breakthrough for cyclohexane is sharper than for benzene, as can be noticed from the steepness of the curves in Figure 5d. This behavior is indicative of preferential interactions with the aromatic guest reasonably due to the formation of host–guest (π–π) interactions.

A similar behavior is observed for COF-300-st (Figure 5e), even though in this case the retention time is of 95 and 83 min/g for benzene and cyclohexane, respectively.

On the other hand, for LZU-111 both benzene and cyclohexane exhibit a similar breakthrough time of ca. 80 min/g. However, after this point, a smoother steep for the benzene curve along with a roll-up for the cyclohexane curve are observed (Figure 5f). This is indicative of replacement of the adsorbed cyclohexane by benzene before complete pore saturation, which proves the slightly stronger interaction of benzene with the LZU-111 pores.

At 323 and 348 K, the retention times of the two vapors are similar (see caption to Figures S15 and S16). Yet, in the case of COF-300-rt and COF-300st, the slope of the curve of cyclohexane after the breakthrough is still slightly steeper than in the case of benzene, highlighting a faster release of the former also at these temperatures. As expected, the higher the temperature, the lower the retention time.

The different behavior of LZU-111 at any temperature can be reasonably ascribed to its framework rigidity vs the flexibility of COF-300, that allows for better interactions among benzene and the pore walls and therefore favors the separation of the two chemicals. In the case of LZU-111, at least in dynamic conditions, the preferential interactions that seem at work among its pore walls and benzene are lower.

Also in the case of the single component breakthrough curves, after the breakthrough, the BEN curve shows a lower steepness than that of CH for COF-300-rt and COF-300-st (Figures S17–19). In any case, benzene is retained for a longer period than cyclohexane.

### Benzene and Cyclohexane Adsorption: Insight by Powder X-ray Diffraction

**Figure 6:**
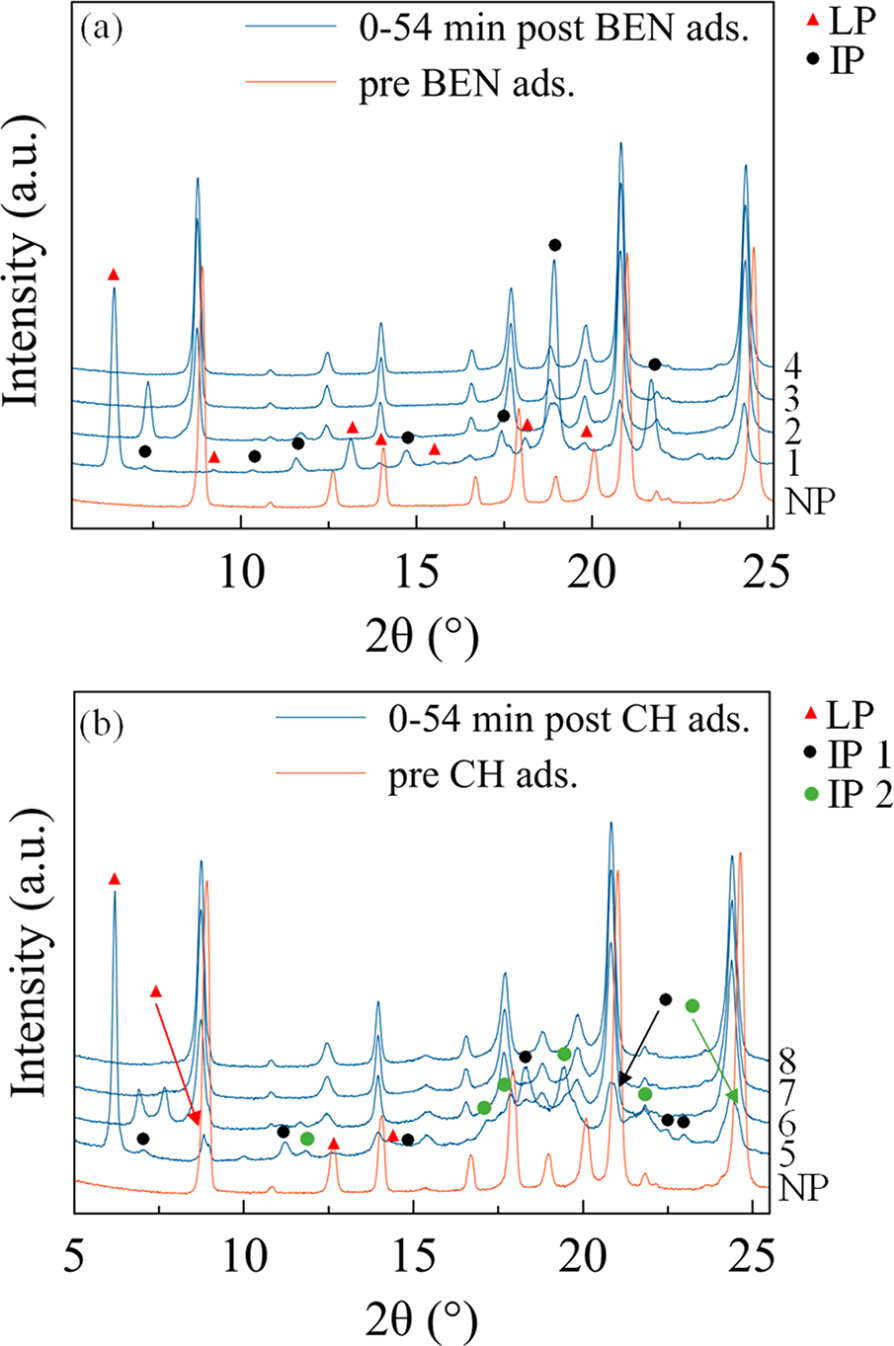
PXRD patterns of COF-300-rt acquired as a function of time, with steps of 18 min, before and after impregnation with (a) benzene (BEN) and (b) cyclohexane (CH). NP, IP, and LP = narrow-pore, intermediate-pore, and larger-pore forms, respectively. 1, NP+IP+LP; 2, NP+IP; 3, NP; 4, NP; 5, NP+IP1+IP2+LP; 6, NP+IP1+IP2; 7, NP; 8, NP. In the case of benzene, the NP form initially disappears and reappears during the acquisition of the first PXRD pattern after impregnation.

As evidenced by Figure 6, COF-300-rt is highly sensitive to benzene and cyclohexane incorporation. Indeed, its PXRD patterns acquired before and after impregnation with benzene and cyclohexane evidence a significant pore aperture with respect to the starting narrow-pore form, confirming the progressive pore aperture suggested by the stepped isotherms (see above).

More in detail, in the PXRD pattern acquired immediately after benzene impregnation (0 min in Figure 6a), a mixture of the narrow-, an intermediate-, and a larger-pore form is present (NP, IP, and LP, respectively, in Figure 6a; see Figure S20 and Table S2 for the details on the data treatment and the unit cell volume of the different forms at each time lapse investigated). Worthy to note, the narrow-pore form is reformed during the acquisition of the first PXRD pattern, as it was not needed to model the first part (5.8–16.0°) of the pattern (evidence of this is the absence of its [020] peak at ca. 9°), but it was needed for the second part.

The larger-pore and intermediate-pore forms have unit cell volumes, respectively, ca. 46–53% and 25–29% larger than the pristine narrow-pore form (Table S1). The larger-pore and intermediate-pore forms gradually return to the narrow-pore form due to benzene desorption. Indeed, 18 min after the impregnation, the larger-pore form has already disappeared, and, after 36 min, also the intermediate-pore form vanishes, leaving the narrow-pore form only. This behavior is a clear evidence of the high plasticity of COF-300 framework. Furthermore, reusability is envisaged.

In the case of cyclohexane, the formation of a larger-pore form and of two different intermediate-pore forms (IP1 and IP2 in Figure 6b) occurred together with the retention of the narrow-pore form, with unit cell volumes, respectively, ca. 48–55%, 18–19%, and 42–44% larger than the pristine narrow-pore form (Table S2). The fact that the narrow-pore form does not temporarily disappear may be considered a proof of the lower affinity of COF-300 for this guest, which does not trigger a complete pore aperture. As with benzene, the desorption of cyclohexane prompted the gradual closure of the pores, and the narrow-pore form is recovered after 36 min. Also in this case, the presence of intermediate-pore and larger-pore forms is consistent with the step-like adsorption isotherms commented above.

The narrow-pore forms recovered at the end of the two experiments have slightly wider channels (Δ*V*/*V* % = 1.7%) than the pristine one. Based on this, the two samples were left on the sample-holder and monitored at different time lapses, up to 42 days (Figure S21a,b, blue traces), i.e., up to an asymptote, at which Δ*V*/*V* % = 0.4%. The pristine form is recovered only upon activation at 393 K for 12 h (Figure S21, green trace).

To shed further light, another set of experiments was carried out on COF-300-rt in the smaller 2θ range 5.5–13.0° (Figure S22). This angular range allowed for a monitoring frequency of about 7 min and confirmed the trend highlighted in Figure 6 (see caption to Figure S22).

To carry out similar impregnation experiments, a batch of COF-300-st was preliminarily activated (393 K, 10^–3^ bar, 12 h). A change in the narrow-pore/larger-pore ratio was observed immediately after benzene or cyclohexane impregnation (Figure S23), even though the low quality of the PXRD data did not allow any treatment. In the case of benzene adsorption, the first peak at *ca*. 6.5° was qualitatively associated with the [020] Bragg reflection of the larger-pore form. As for cyclohexane adsorption, the same peak is present with lower integrated intensity and concomitant with that of the pristine narrow-pore form (8.6°). Also in this case, the cyclohexane does not completely open the NP form present. With both vapors, 18 min after the impregnation the pristine mixture is restored. As regards COF-300-st response to benzene, the impregnation and vapor adsorption experiments show a slightly different behavior. Indeed, when impregnated, COF-300-st does not reach a complete pore aperture, while the large hysteresis in the desorption branch of the isotherm suggests high host–guest affinity. This occurrence could be explained by the different experimental setup. The PXRD impregnation experiments have been carried out in air (in the presence of interferents), at room temperature, in nonequilibrium conditions, and the two solvents are in liquid form and not as vapors.

**Figure 7:**
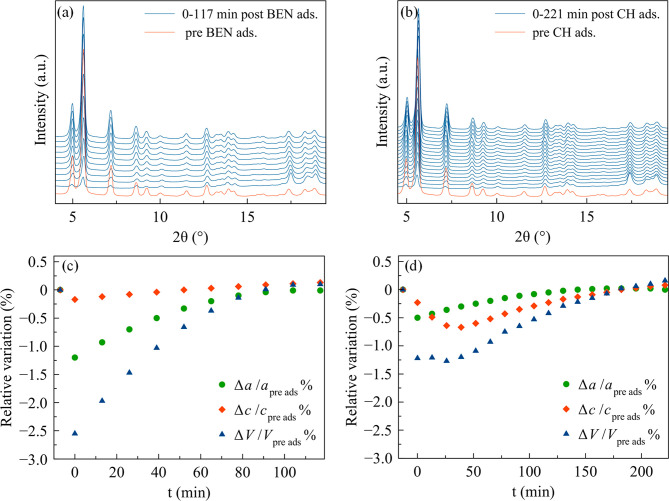
PXRD patterns of LZU-111 as a function of time, with steps of 13 min, before and after impregnation with (a) benzene (BEN) and (b) cyclohexane (CH). The loss of crystallinity immediately after the impregnation is due to the copresence of a liquid phase. Percentage relative variation of the unit cell parameters of LZU-111 after impregnation with (c) benzene and (d) cyclohexane normalized with respect to the preadsorption values.

Finally, as expected, in the case of activated (393 K and 10^–3^ bar for 12 h), LZU-111 impregnation with benzene and cyclohexane (Figure 7a,b, respectively) triggers a different behavior with respect to that of COF-300, as no pore aperture occurs. As unveiled by a whole powder pattern parametric refinement (Figure S24a,b, respectively, and Tables S3 and S4), the framework undergoes a limited contraction (highest Δ*V*/*V* % with benzene = −2.6%, Figure 7c; highest Δ*V*/*V* % with cyclohexane = −1.3%, Figure 7d), which might be due to the insurgence of host–guest interactions narrowing the pores. Worthy of note, the unit cell volume variation of COF-300 is about 20 times that of LZU-111.

## Conclusions

This work has highlighted the impact of pore flexibility for the adsorption and separation of benzene and cyclohexane, a still underexplored functional application in the field of COFs. In the case of COF-300, benzene and cyclohexane isotherms are characterized by steps, indicative of guest-induced plasticity. Moreover, COF-300 isotherms show large hysteresis loops, which are not closed with benzene, suggesting the insurgence of preferential (π–π) host–guest interactions inhibiting the complete vapor release.

*In situ* PXRD experiments on benzene- and cyclohexane-impregnated batches are indicative of high flexibility for COF-300, exhibiting narrow, intermediate and large pore forms, trapped in sequence for the first time. These observations nicely rationalize COF-300 stepped and hysteretic benzene and cyclohexane isotherms. By contrast, LZU-111 does not exhibit any noticeable guest-induced form change.

Two different scenarios were observed also in dynamic conditions: LZU-111 exhibits a low selectively for benzene over cyclohexane, while COF-300 is able to separate them, as it is evidenced by the longer breakthrough of benzene, demonstrating the higher adaptability of its flexible framework. Summarizing, the different framework flexibility translates into a higher selectivity of COF-300 vs LZU-111 in the separation of benzene from cyclohexane.

## Supporting Information Available

Discussions of synthesis of TAM (1) and calculation of the BET specific surface areas, figures of ^1^H and ^13^C NMR spectra of 1a in CDCl_3_, ^1^H and ^13^C NMR spectra of 1b in DMSO-*d*_6_, ^1^H and ^13^C NMR spectra of 1 in DMSO-*d*_6_, IR spectra of COF-300-rt, COF-300-st, and LZU-111, SEM images of samples of COF-300-rt, COF-300-st, and LZU-111, crystal size distribution in COF-300-rt, COF-300-st, and LZU-111 samples, PXRD data treatment of COF-300-rt and LZU-111 during the VT-PXRD experiment, VT-PXRD experiment and data treatment on COF-300-rt and COF-300-st, powder patterns of COF-300-rt and COF-300-st before and after thermal activation, pore-size distribution for COF-300-rt, COF-300-st, and LZU-111, breakthrough curves of COF-300-rt, COF-300-st, and LZU-111 flowing a 50:50 v/v mixture of benzene and cyclohexane at 323 and 348 K, breakthrough curves of COF-300-rt, COF-300-st, and LZU-111 flowing benzene and cyclohexane at 298, 323, and 348 K, PXRD data treatment after impregnation of COF-300-rt with benzene and cyclohexane, PXRD monitoring of COF-300-rt after the impregnation experiments, PXRD monitoring of COF-300-rt after impregnation with benzene and cyclohexane, PXRD monitoring of COF-300-st after impregnation with benzene and cyclohexane, PXRD data treatment after impregnation of LZU-111 with benzene and cyclohexane, and tables of details of the PXRD data treatment after impregnation of COF-300-rt with benzene and cyclohexane and after impregnation of LZU-111 with benzene and cyclohexane (PDF)
