# Supplementary material for: Impact of Pore Flexibility in Imine-Linked Covalent Organic Frameworks on Benzene and Cyclohexane Adsorption
Source: ACS Appl Mater Interfaces. 2022 Aug 30;14(36):40890–901. doi: 10.1021/acsami.2c09911 (PMC9931175; doi:10.1021/acsami.2c09911)
Supplement: Supplementary file 1 — am2c09911_si_001.pdf [file am2c09911_si_001.pdf]

Supplementary Information for

IMPACT OF PORE FLEXIBILITY IN IMINE-LINKED COVALENT ORGANIC FRAMEWORKS ON BENZENE AND CYCLOHEXANE ADSORPTION

Marco Moroni,[a] Esther Roldan-Molina,[b,c] Rebecca Vismara,[a,b*]

Simona Galli,[a,d*] Jorge A.R. Navarro[b*]

[a]Dipartimento di Scienza e Alta Tecnologia, Università dell'Insubria

Via Valleggio 11, 22100, Como, Italy

[b]Departamento de Química Inorgánica, Universidad de Granada

Avenida de Fuentenueva S/N, 18071, Granada, Spain

[c]Instituto de Investigaciones Químicas, CSIC-Universidad de Sevilla,

Calle Américo Vespucio 49, 41092 Seville, Spain

[d]Consorzio Interuniversitario Nazionale per la Scienza e Tecnologia dei Materiali

50121 Firenze, Italy

Corresponding authors: SG, simona.galli@uninsubria.it; JARN, jarn@ugr.es; RV, rvismara@ugr.es

Summary

Section S1. Synthesis of tetrakis(4-aminophenyl)methane (TAM) (1)

S1.1. Synthesis of tetraphenylmethane (1a) S4

S1.2. Synthesis of tetrakis(4-nitrophenyl)methane (1b) S4

S1.3. Synthesis of tetrakis(4-aminophenyl)methane (1) S4

Section S2. Figures

Figure S1 S6

Figure S2 S6

Figure S3 S7

Figure S4 S7

Figure S5 S8

Figure S6 S8

Figure S7 S9

Figure S8 S10

Figure S9 S11

Figure S10 S11

Figure S11 S12

Figure S12 S13

Figure S13 S14

Figure S14 S15

Figure S15 S16

Figure S16 S17

Figure S17 S18

Figure S18 S19

Figure S19 S20

Figure S20 S21

Figure S21 S22

Figure S22 S23

Figure S23 S24

Figure S24 S24

Section S3. Tables

Table S1 S25

Table S2 S25

Table S3 S26

Table S4 S27

Section S4. Calculation of the BET specific surface areas S28

Section S5. References S29

Section S1. Synthesis of tetrakis(4-aminophenyl)methane (TAM) (1)

S1.1. Synthesis of tetraphenylmethane (1a)

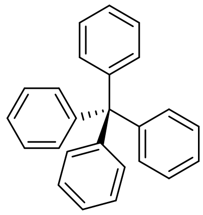

Compound **1a** was synthetized following a procedure described in the literature.[1] ^{1}H NMR (500 MHz, $CDCl_3$, Figure S1) δ (ppm) 7.25–7.17 (m, 20H). ^{13}C NMR (125 MHz, $CDCl_3$, Figure S2) δ (ppm): 146.8, 131.2, 127.5, 125.9, 65.0. The physical and spectroscopic properties agreed with those reported in the literature.[2]

S1.2. Synthesis of tetrakis(4-nitrophenyl)methane (1b)

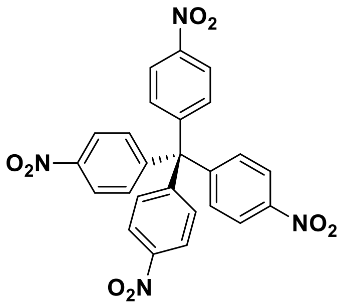

Compound **1b** was synthetized following a procedure described in the literature.[2] ^{1}H NMR (500 MHz, DMSO-d_6, Figure S3) δ (ppm): 8.23 (d, J = 9.0 Hz, 8H), 7.60 (d, J = 9.0 Hz, 8H). ^{13}C NMR (125 MHz, DMSO-d_6, Figure S4) δ (ppm): 151.5, 146.6, 132.0, 124.3, 65.8. The physical and spectroscopic properties agreed with those reported in the literature.[3]

S1.3. Synthesis of tetrakis(4-aminophenyl)methane (1)

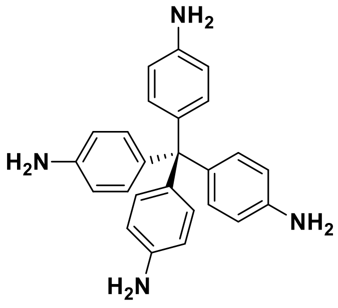

Compound **1** was prepared using a modified version of Bräse's literature procedure.[2] In a round bottom flash tetrakis(4-nitrophenyl)methane **1b** (5.00 g, 9.99 mmol) and Pd/C (5%, 1.00 g, 0.47 mmol) were suspended in methanol (200 mL). The reaction mixture was bubbled with H_2 for 10 min. After this, the reaction mixture was vigorously stirred under H_2 atmosphere at room temperature for 24 h Then, the mixture was filtered through a plug of Celite and rinsed with methanol and tetrahydrofuran. Finally, the filtrate was concentrated in vacuum and the crude was purified by flash column chromatography (dichloromethane/methanol 7:1) to yield **1** as a yellowish solid (3.08 g, 8.1 mmol, yield 81%). ^{1}H NMR (500 MHz, DMSO-d_6, Figure S5) δ (ppm): 6.67 (d, J = 8.7 Hz, 8H), 6.39 (d, J = 8.7 Hz, 8H), 4.85 (br s, 8H) ppm. ^{13}C NMR (125 MHz, DMSO-

d_6, Figure S6) δ (ppm): 146.1, 136.3, 131.5, 113.1, 61.6. The physical and spectroscopic properties agreed with those reported in the literature.[3]

Section S2. Figures

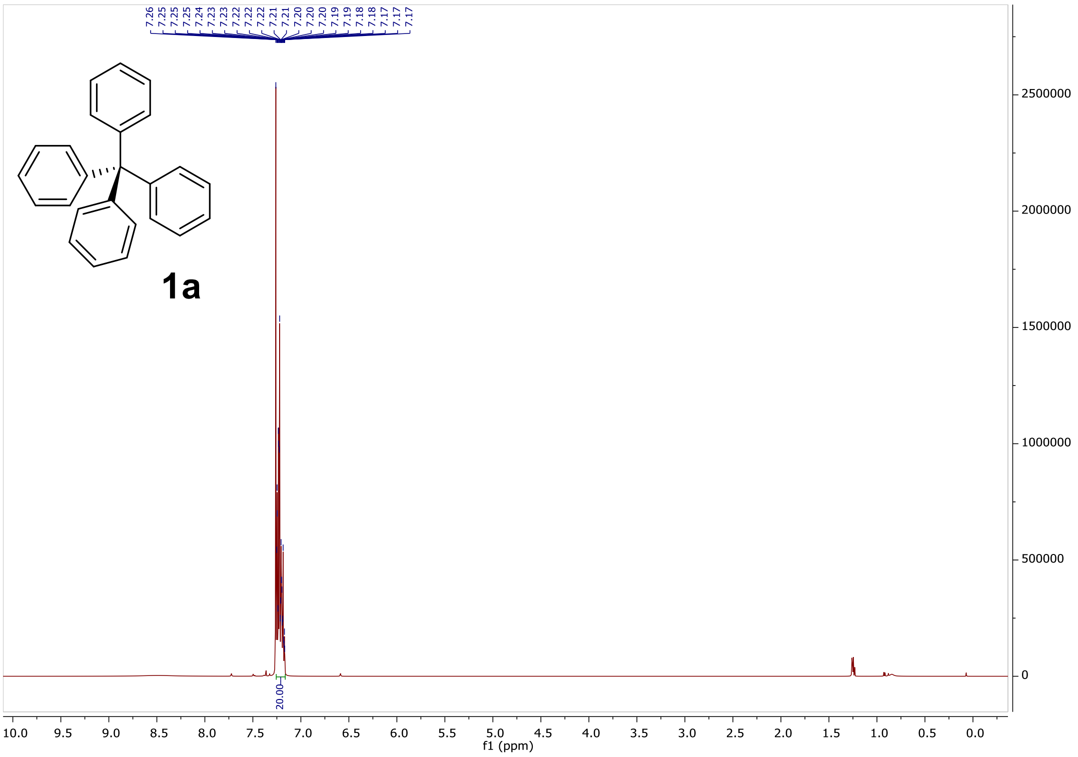

Figure S1. ^{1}H NMR spectrum of **1a** in $CDCl_3$.

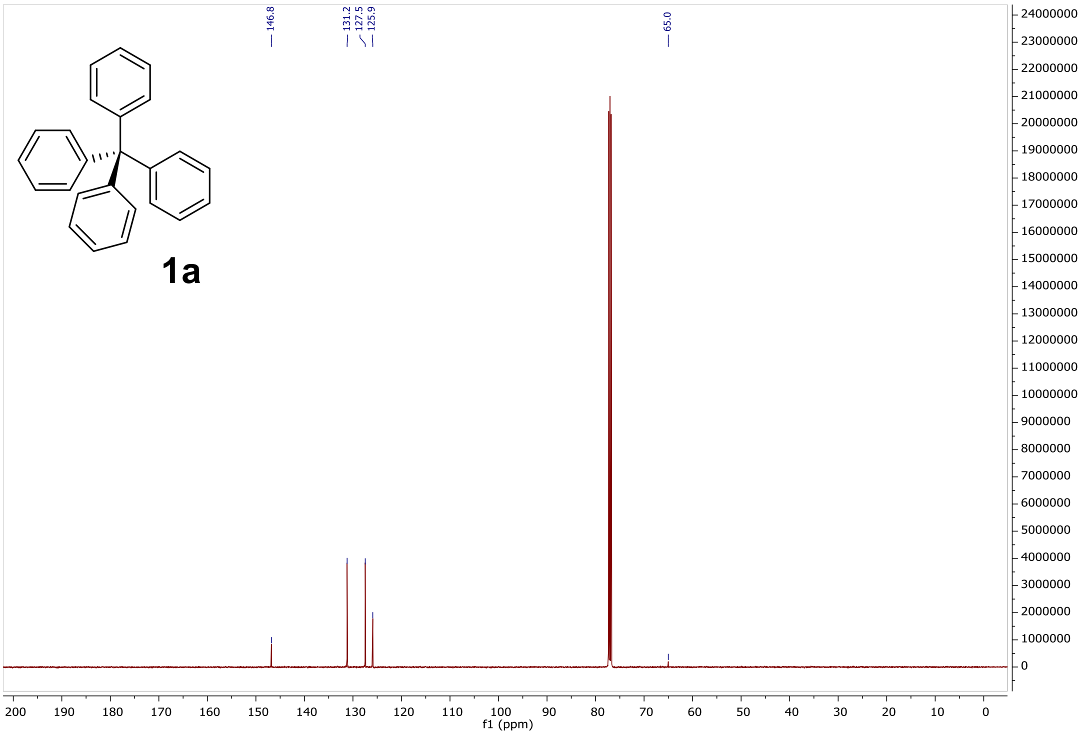

Figure S2. ^{13}C NMR spectrum of **1a** in $CDCl_3$.

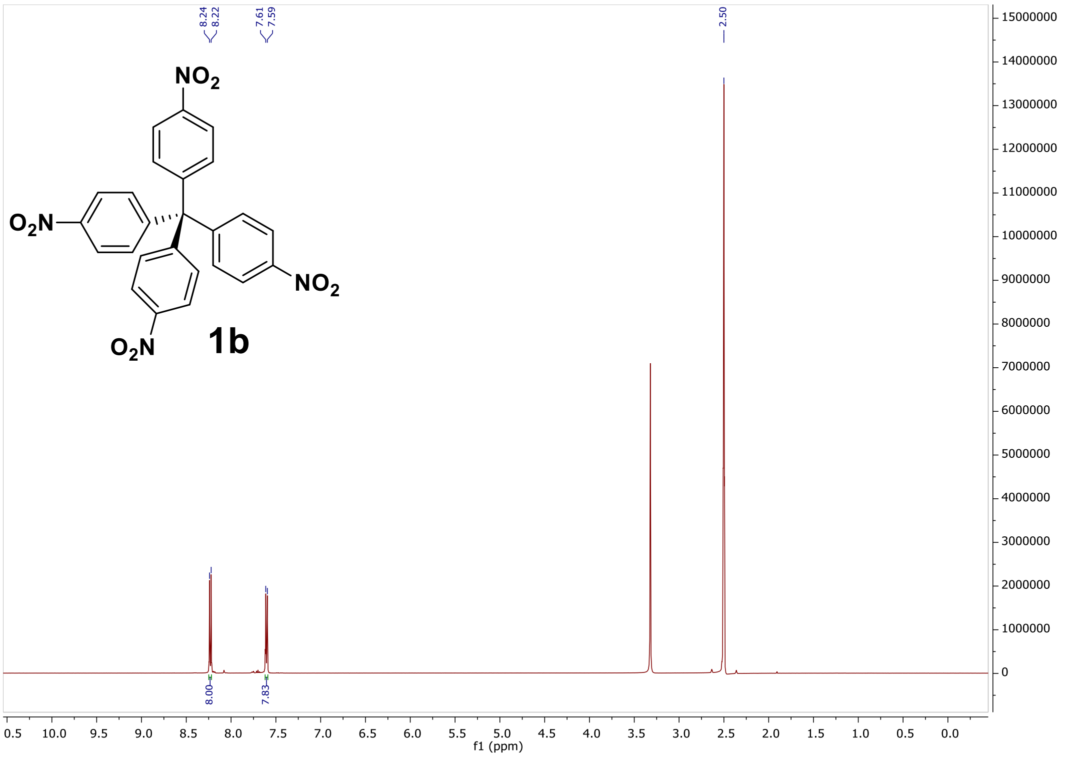

Figure S3. ^{1}H NMR spectrum of **1b** in DMSO-d_6.

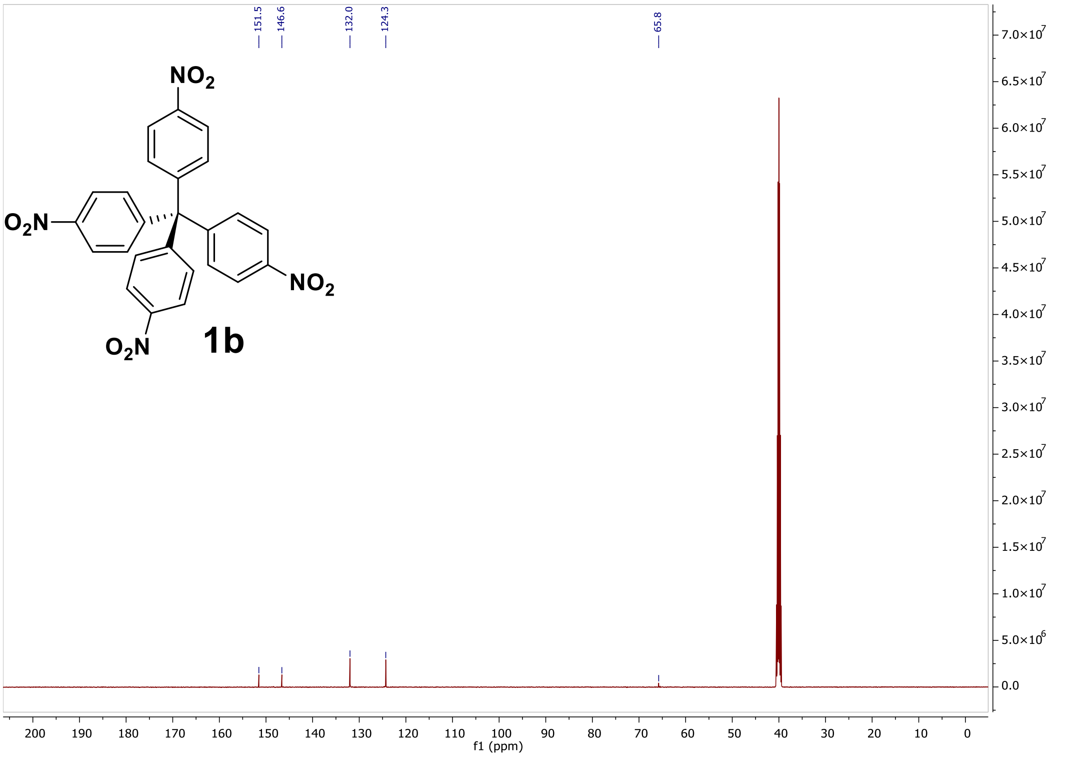

Figure S4. ^{13}C NMR spectrum of **1b** in DMSO-d_6.

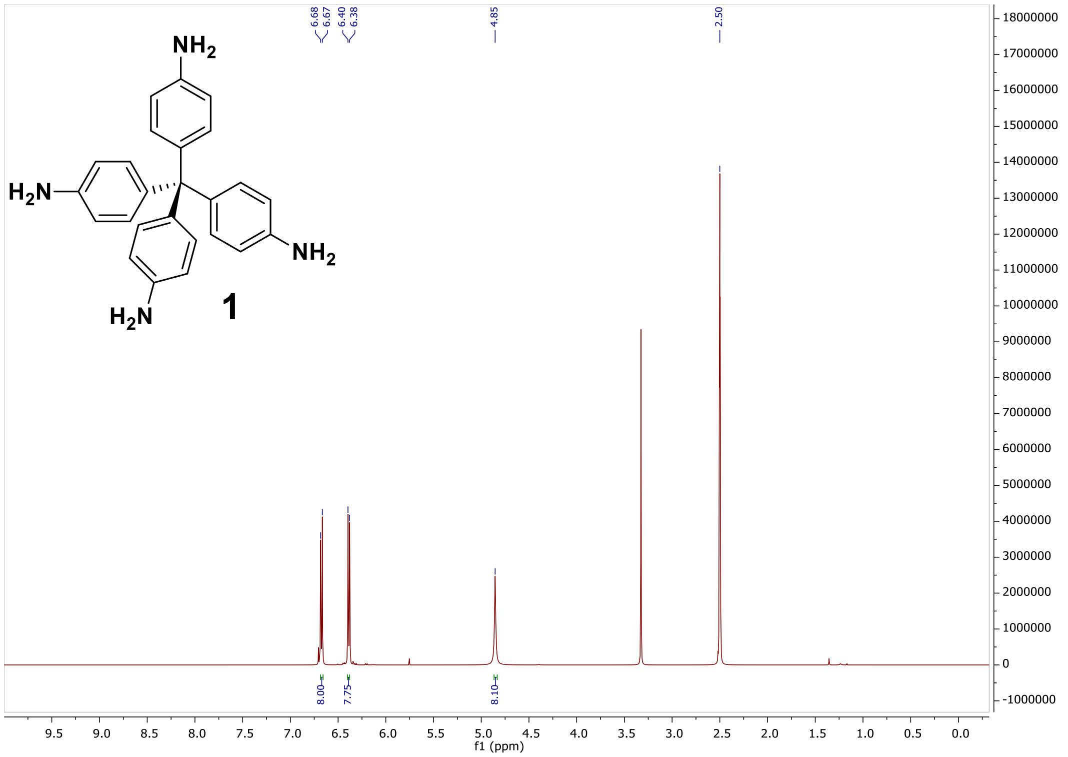

Figure S5. ^{1}H NMR spectrum of **1** in DMSO-d_6.

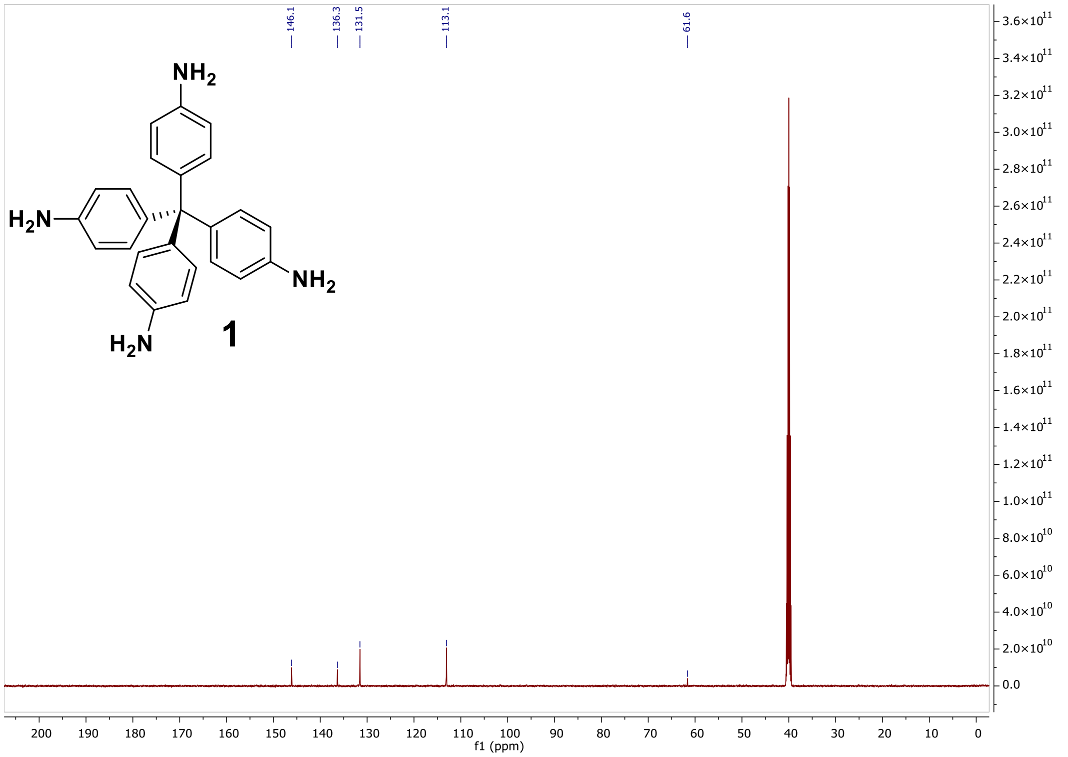

Figure S6. ^{13}C NMR spectrum of **1** in DMSO-d_6.

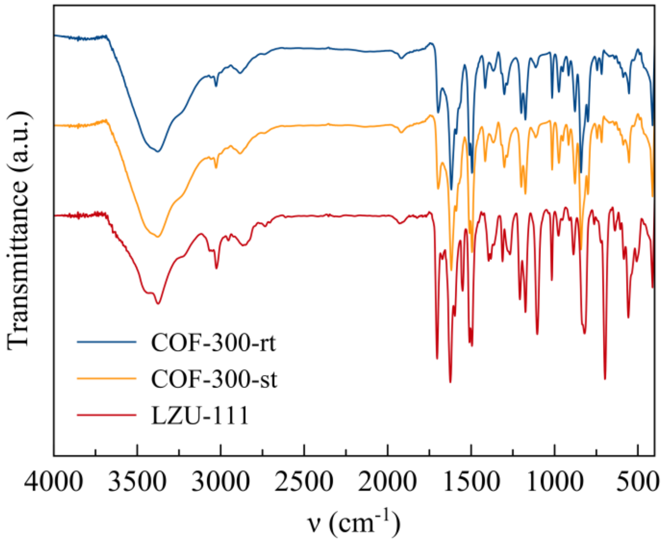

Figure S7. IR spectra of COF-300-rt, COF-300-st and LZU-111.

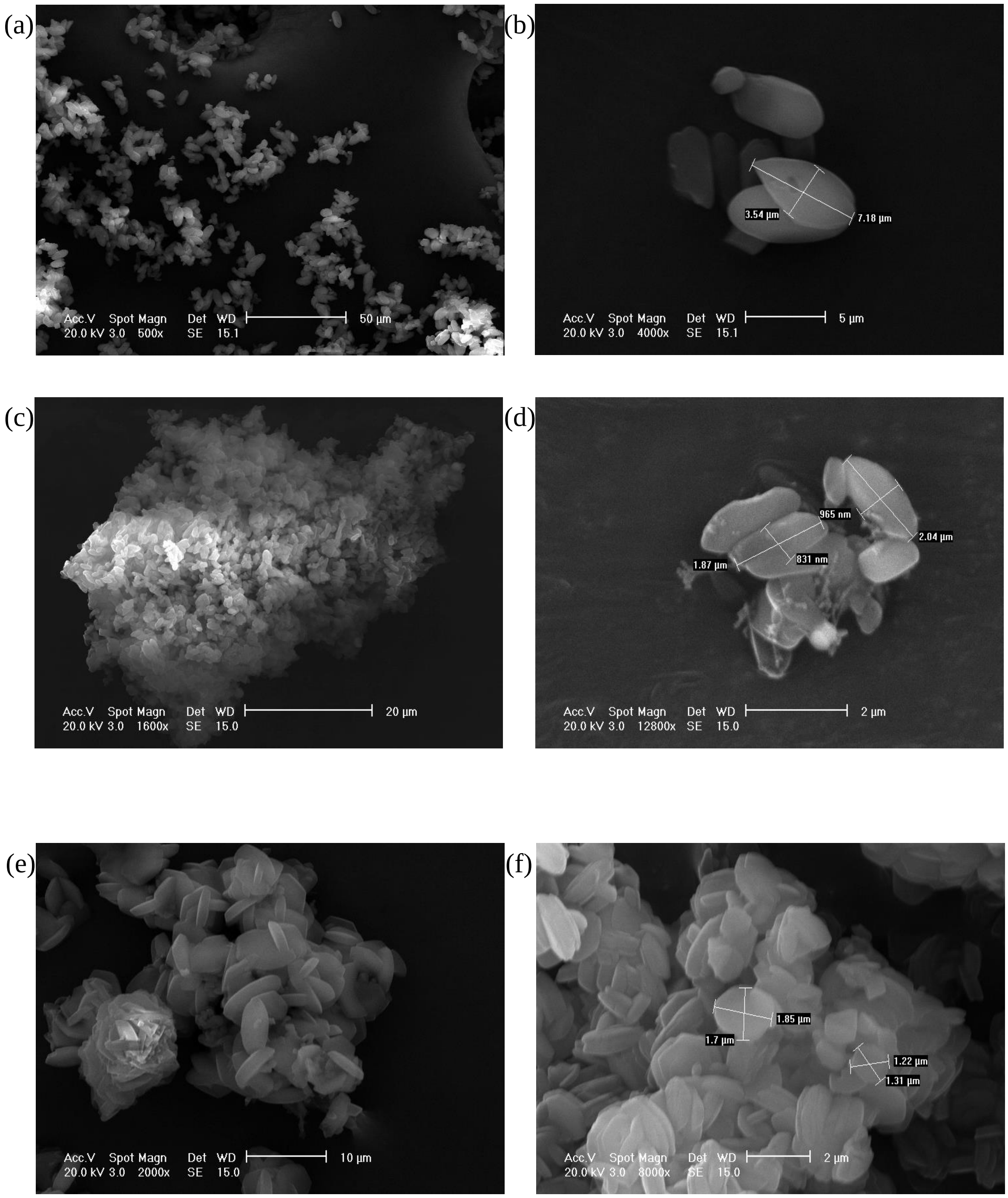

Figure S8. SEM images of (a, b) COF-300-rt, (c, d) COF-300-st and (e, f) LZU-111.

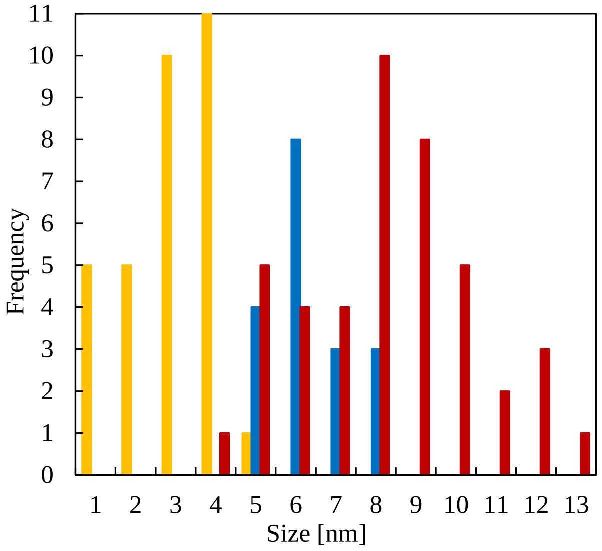

Figure S9. Statistical crystal size distribution for a) COF-300-rt (blue), corresponding to the SEM images in Figures 2b of the main text and S8a,b; b) COF-300-st (orange), corresponding to the SEM images in Figures 2d of the main text and S8c,d; c) LZU-111 (dark red), corresponding to the SEM images in Figures 2f of the main text and S8e,f.

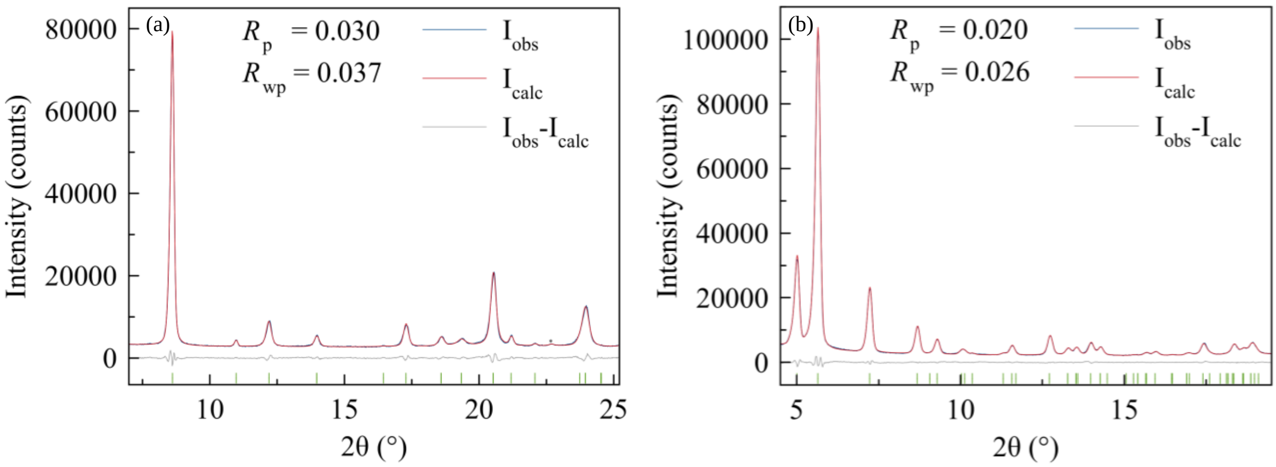

Figure S10. Graphical representation of the whole powder pattern parametric refinements carried out on the data acquired at (a) 378 K on COF-300-rt and (b) 338 K on LZU-111, as a representative example of the data treatment. Observed, calculated and difference patterns: blue, red and grey traces, respectively. The ticks at the bottom indicate the position of the Bragg reflections.

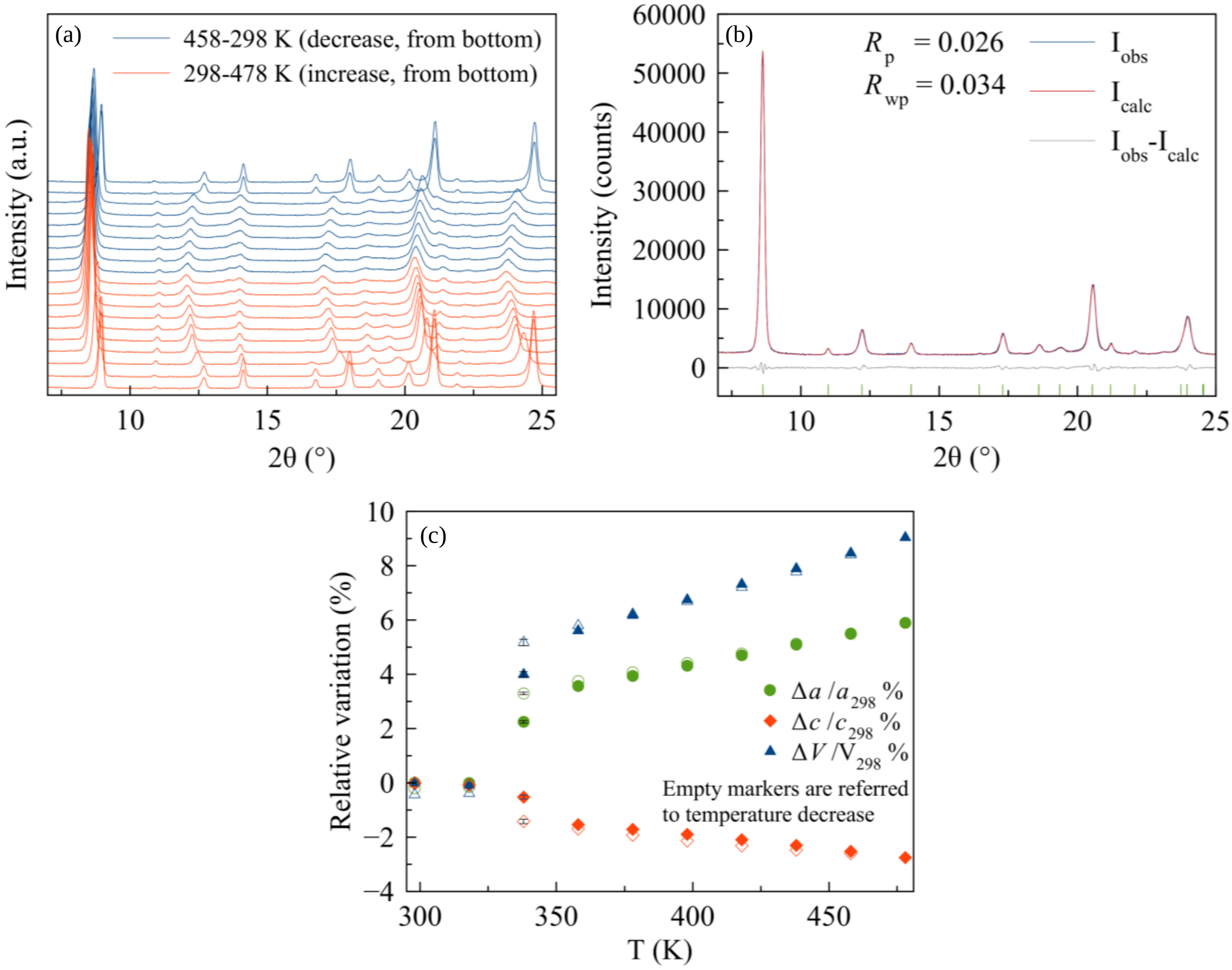

Figure S11. (a) Powder X-ray diffraction patterns of COF-300-rt acquired as a function of the temperature. From the bottom: from 298 to 478 K with steps of 20 K, red traces; from 458 to 298 K with steps of 20 K, blue traces. (b) Graphical representation of the whole powder pattern parametric refinement carried out on the data acquired at 378 K, as a representative example of the data treatment. Observed, calculated and difference patterns: blue, red and grey traces, respectively. The ticks at the bottom indicate the position of the Bragg reflections. (c) Percentage relative variation of the unit cell parameters, normalized with respect to the values at 298 K, as a function of the temperature. Empty symbols describe the cooling branch. To confirm the existence of hysteresis, error bars are present on the values estimated at 338 K.

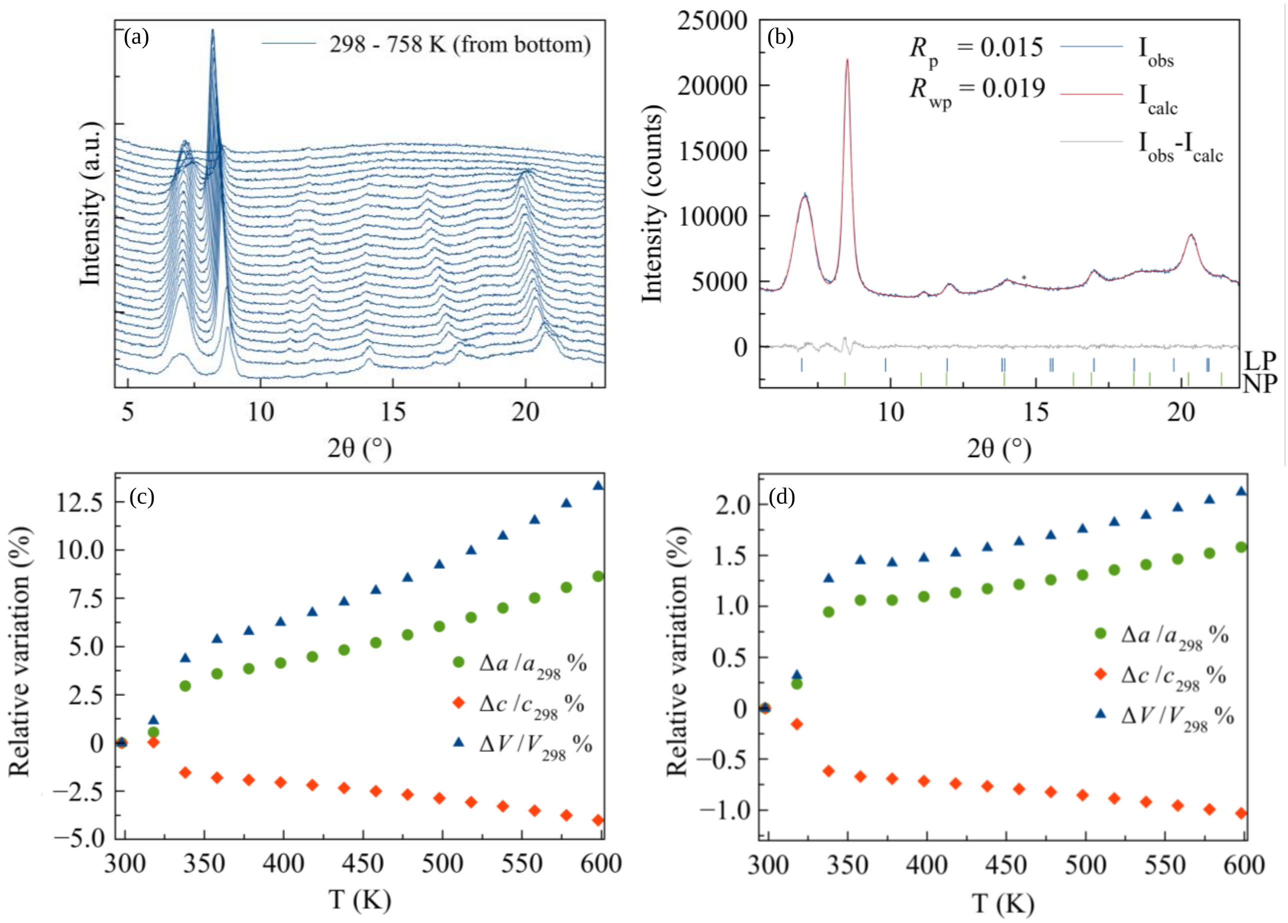

Figure S12. (a) Powder X-ray diffraction patterns of COF-300-st acquired as a function of the temperature. From the bottom: 298 K to 758 K with steps of 20 K. (b) Graphical representation of the whole powder pattern refinement carried out on the data acquired at 378 K, as a representative example of the data treatment. Observed, calculated and difference patterns: blue, red and grey traces, respectively. The ticks at the bottom indicate the position of the Bragg reflections. The asterisk indicates a peak belonging to an impurity. (c), (d) Percentage relative variation of the narrow-pore form (c) and the larger-pore form (d) unit cell parameters, normalized with respect to the values at 298 K, as a function of the temperature.

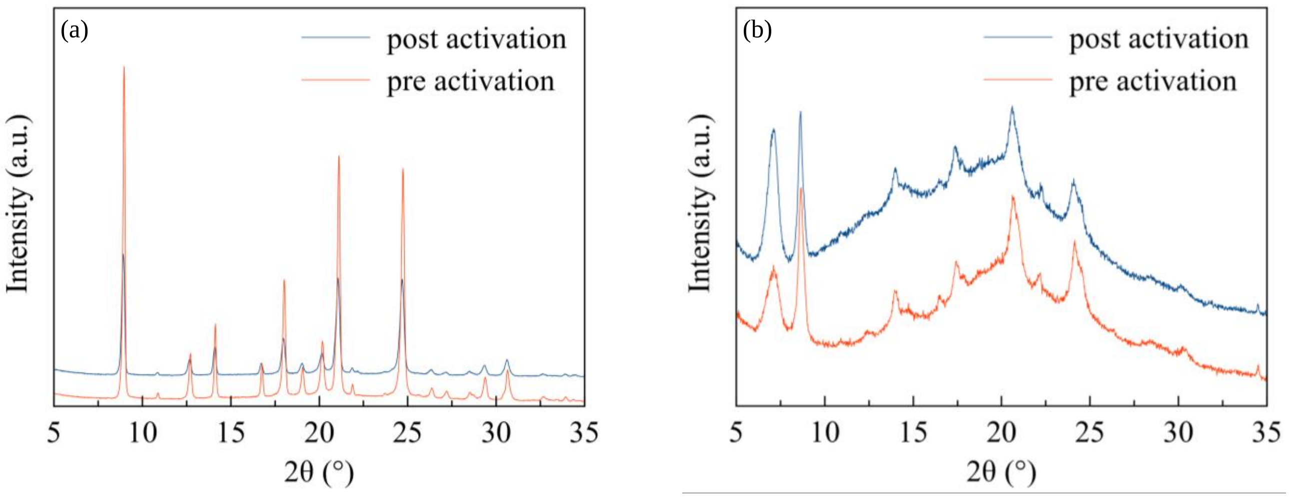

Figure S13. Powder X-ray diffraction patterns of (a) COF-300-rt and (b) COF-300-st before and after thermal activation.

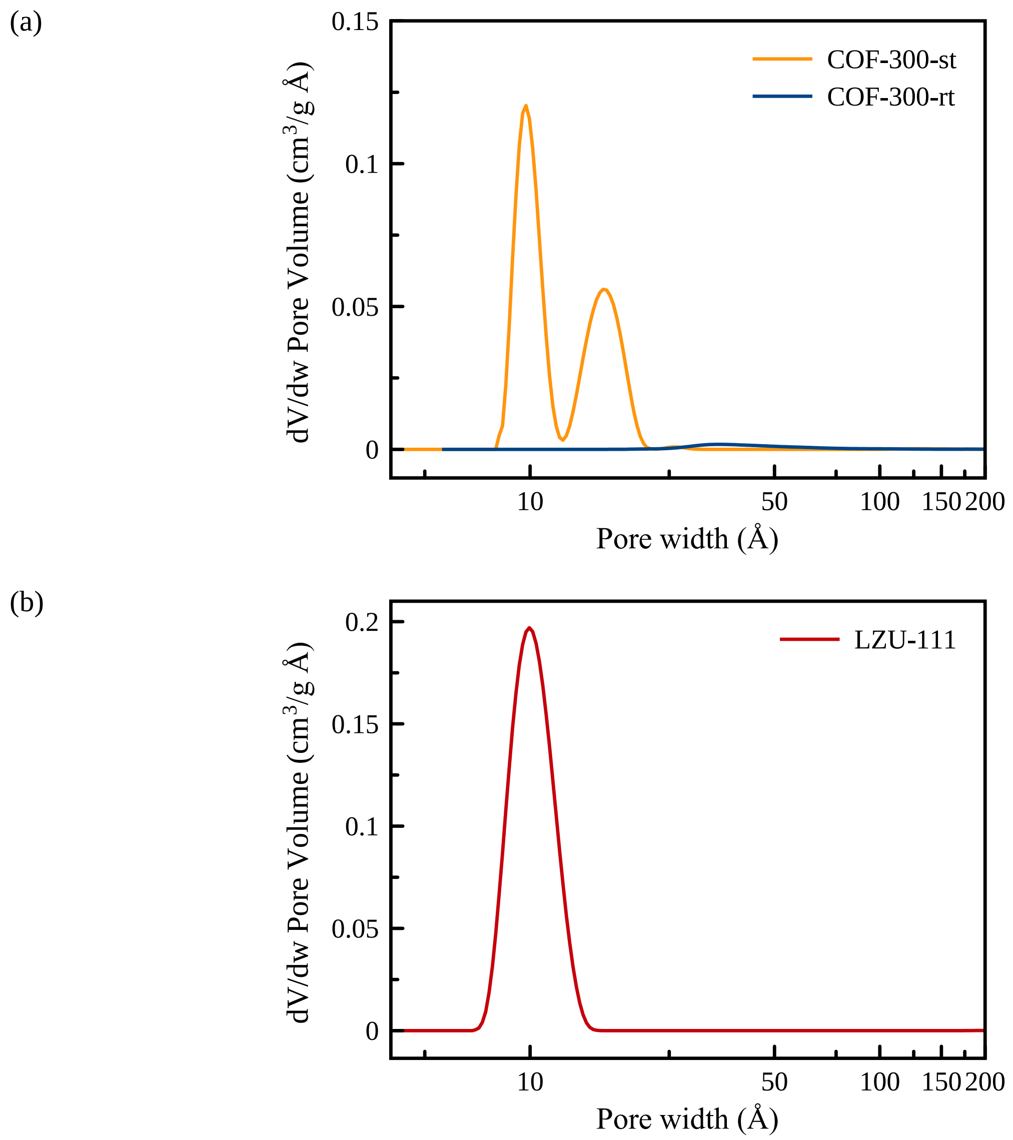

Figure S14. Pore distribution of (a) COF-300-rt and COF-300-st and (b) LZU-111 retrieved from the N_2 adsorption isotherms at 77 K by the 2D-NLDFT method.[4]

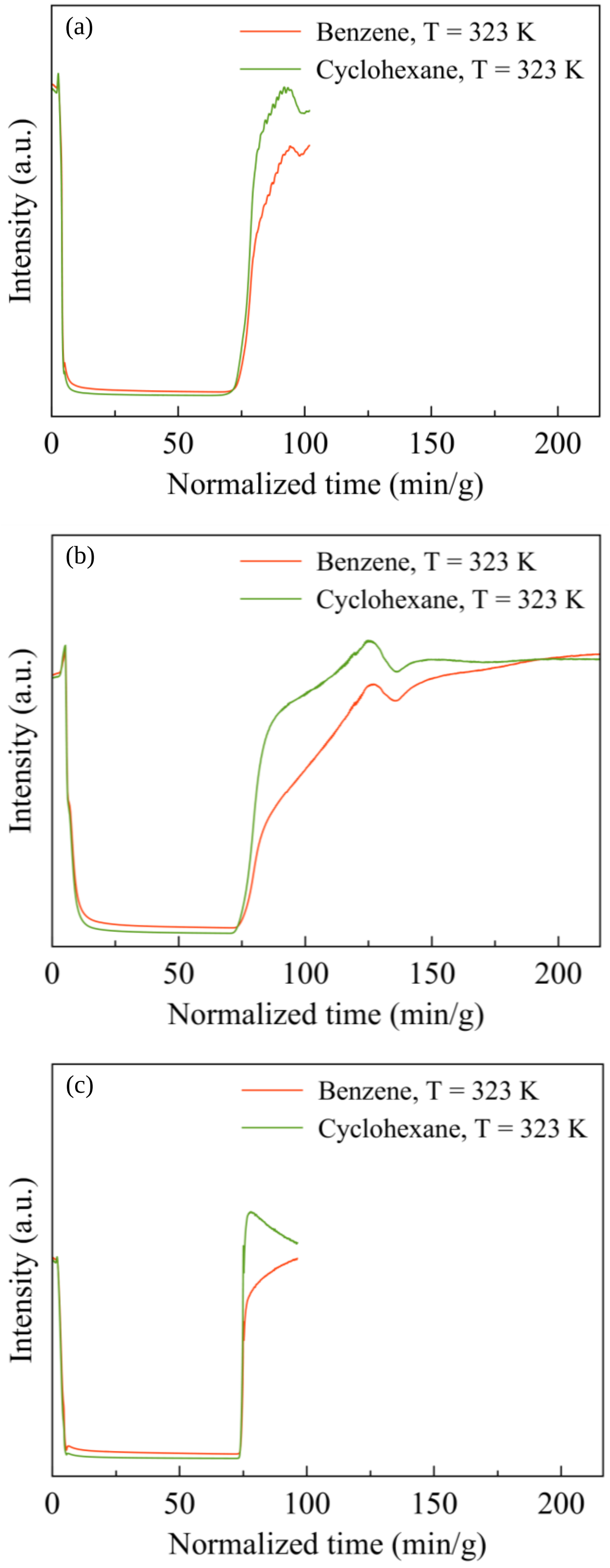

Figure S15. Breakthrough curves of (a) COF-300-rt, (b) COF-300-st and (c) LZU-111 flowing a 50:50 v/v mixture of benzene and cyclohexane at 323 K. Breakthrough times (benzene/cyclohexane): (a) 66/62 min/g; (b) 71/70 min/g; (c) 72/72 min/g. The horizontal axis has been set to allow a straightforward comparison with Figure 5.

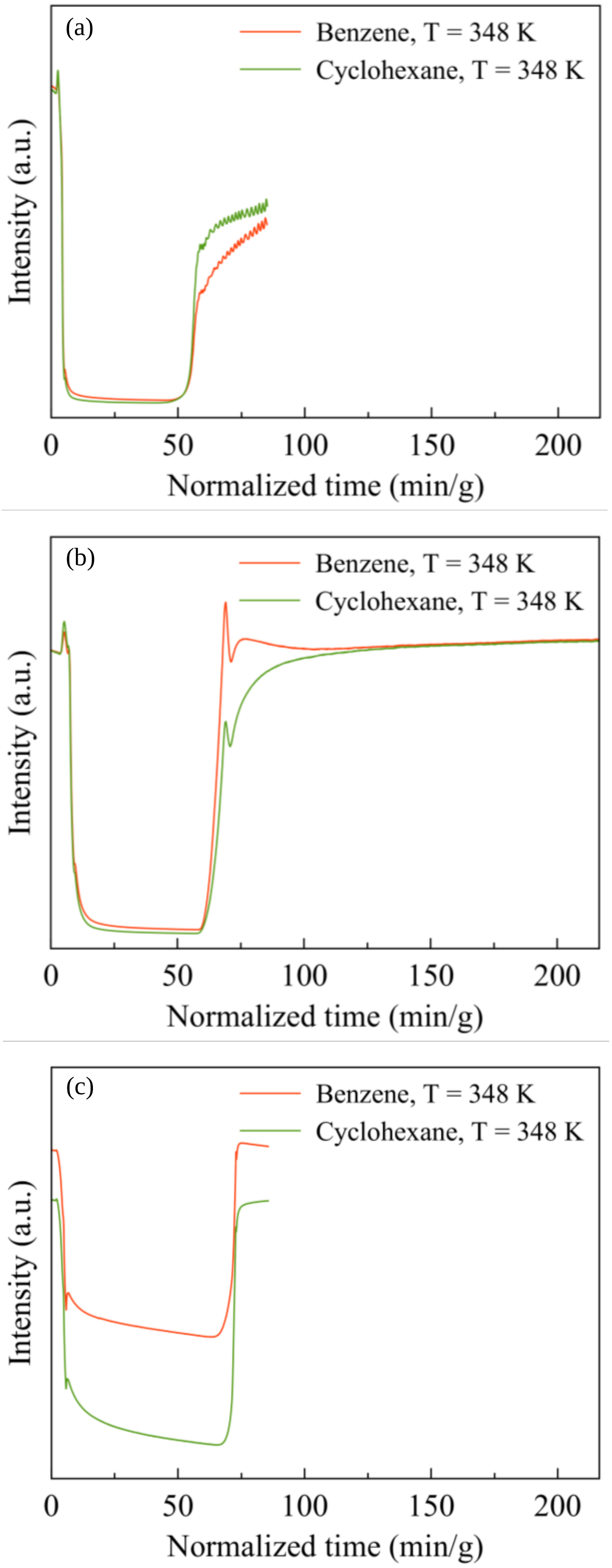

Figure S16. Breakthrough curves of (a) COF-300-rt, (b) COF-300-st and (c) LZU-111 flowing a 50:50 v/v mixture of benzene and cyclohexane at 348 K. Breakthrough times (benzene/cyclohexane): (a) 45/40 min/g; (b) 58/57 min/g; (c) 64/65 min/g. The horizontal axis has been set to allow a straightforward comparison with Figure 5.

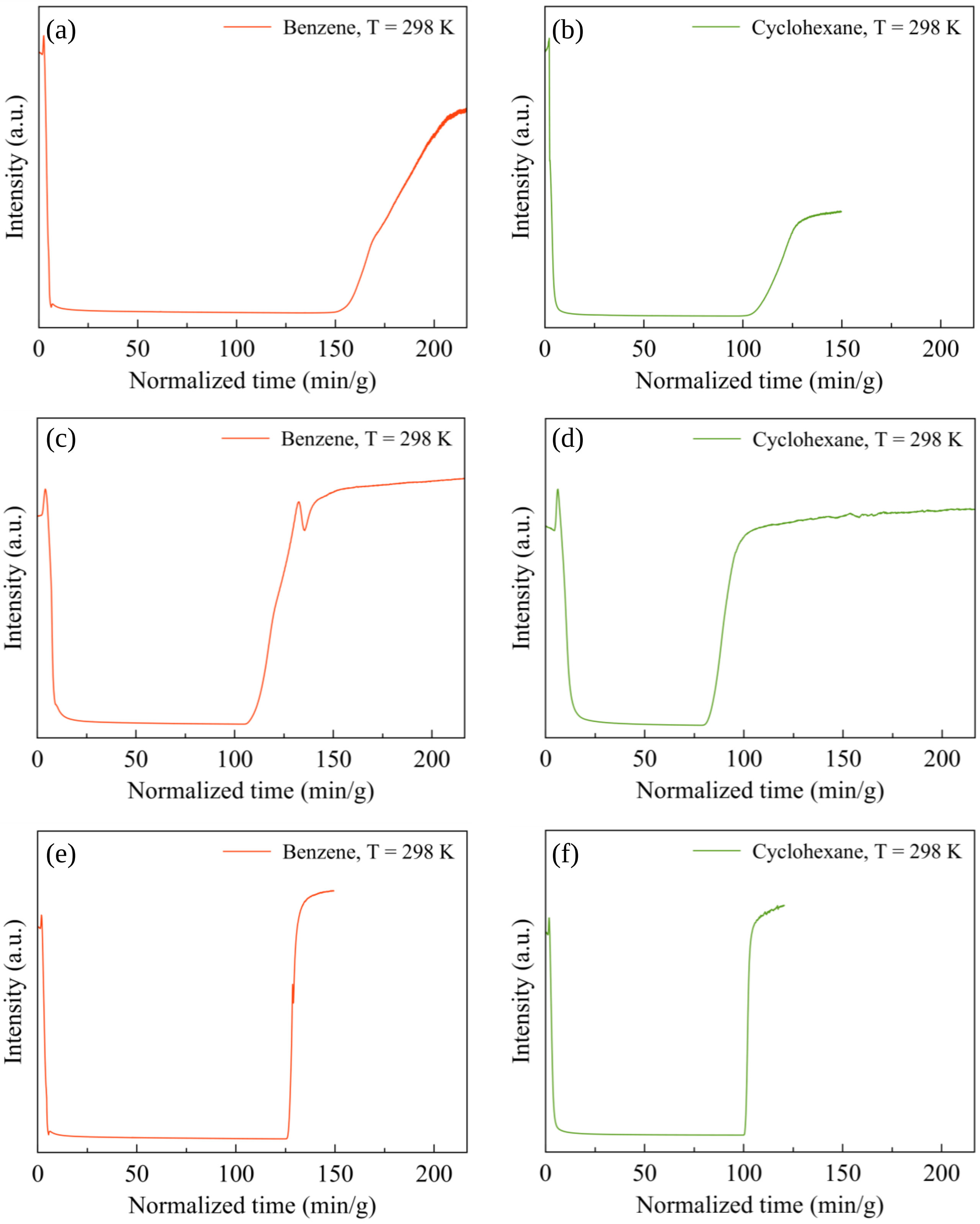

Figure S17. Breakthrough curves of (a, b) COF-300-rt, (c, d) COF-300-st and (e, f) LZU-111 flowing benzene (a, c, e) and cyclohexane (b, d, f) at 298 K. The horizontal axis has been set to allow a straightforward comparison with Figure 5.

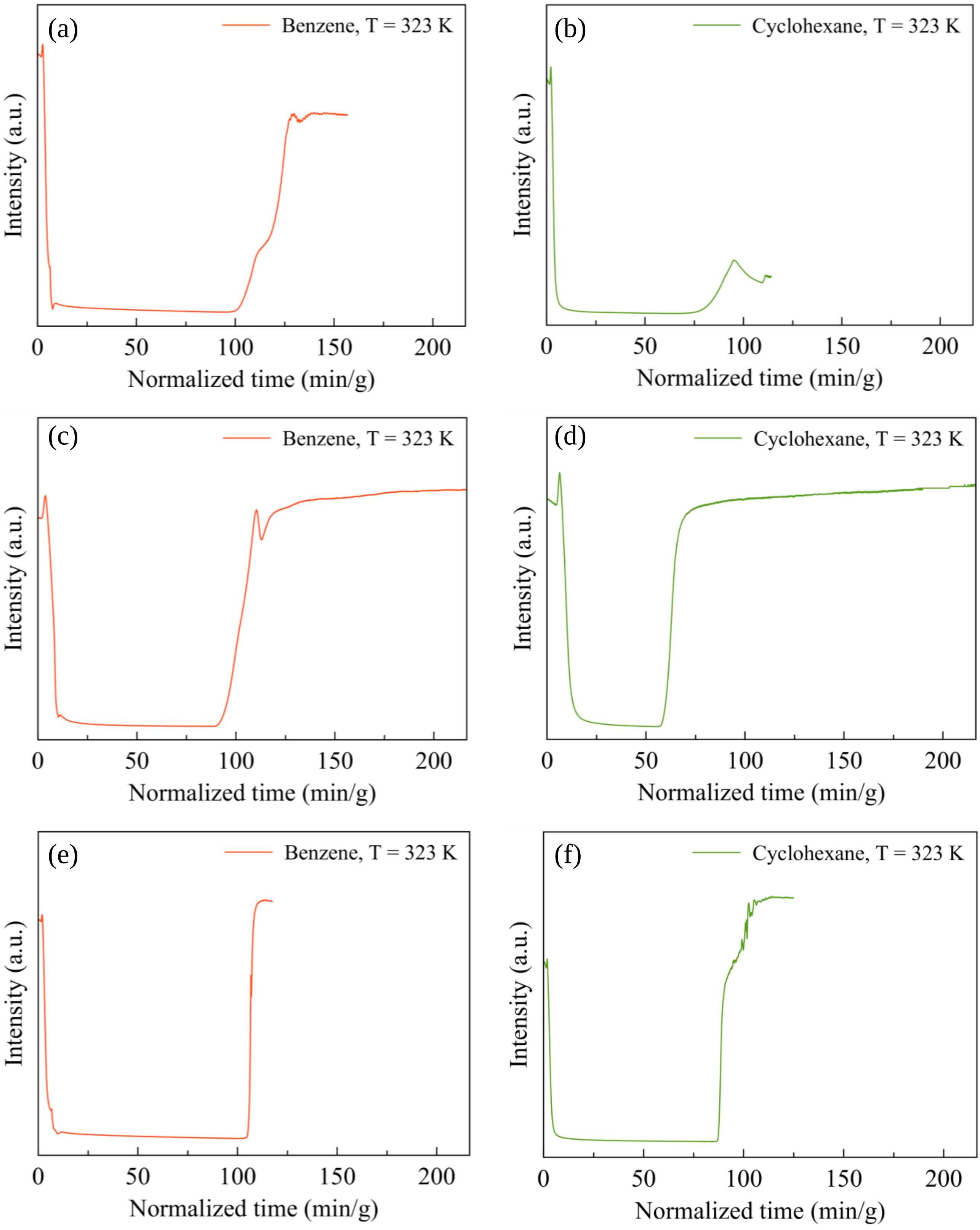

Figure S18. Breakthrough curves of (a, b) COF-300-rt, (c, d) COF-300-st and (e, f) LZU-111 flowing benzene (a, c, e) and cyclohexane (b, d, f) at 323 K. The horizontal axis has been set to allow a straightforward comparison with Figure 5.

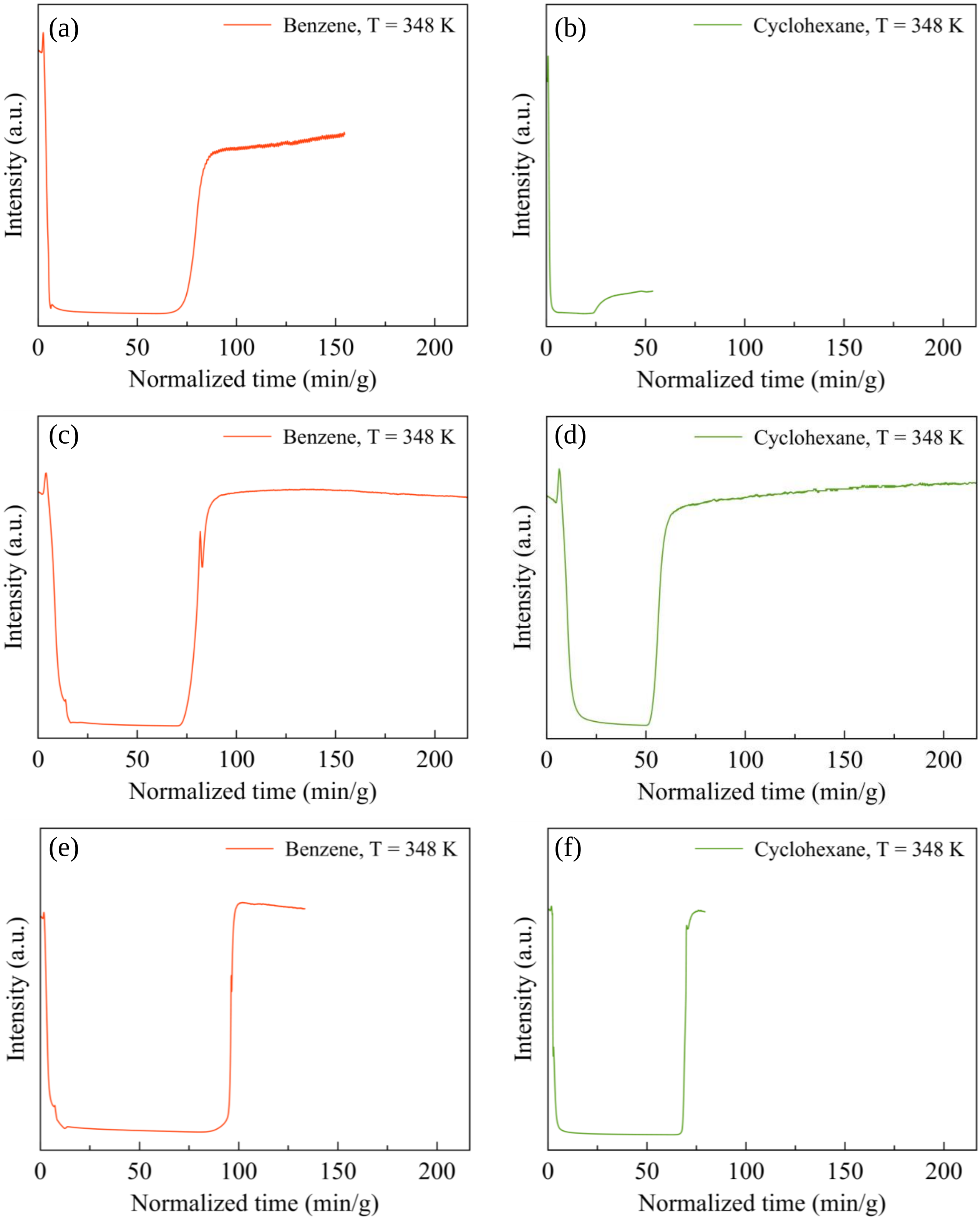

Figure S19. Breakthrough curves of (a, b) COF-300-rt, (c, d) COF-300-st and (e, f) LZU-111 flowing benzene (a, c, e) and cyclohexane (b, d, f) at 348 K. The horizontal axis has been set to allow a straightforward comparison with Figure 5.

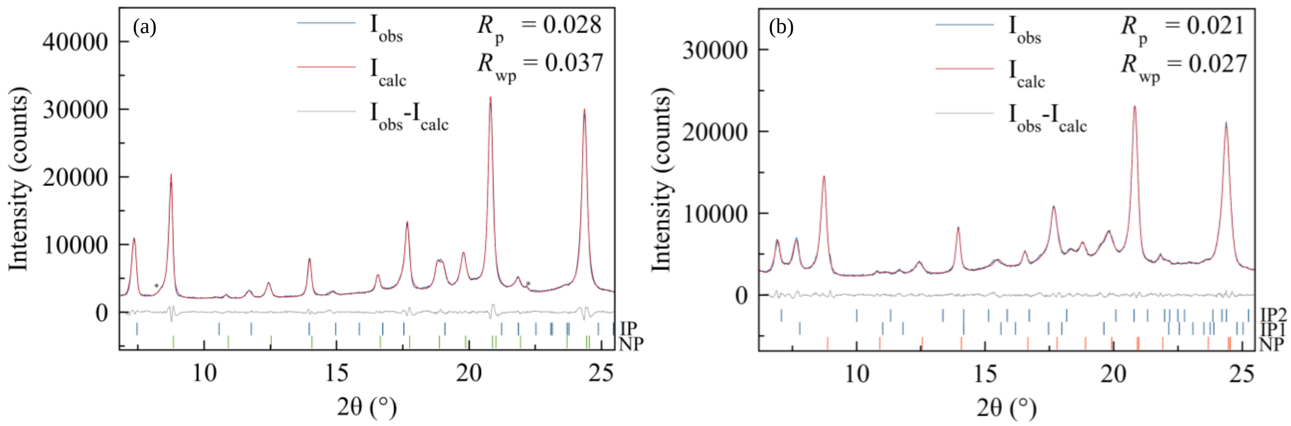

Figure S20. Graphical representation (as a representative example) of the whole powder pattern refinements carried out on the data of COF-300-rt acquired 18 minutes after the impregnation with (a) benzene and (b) cyclohexane. Observed, calculated and difference patterns: blue, red and grey traces, respectively. The ticks at the bottom indicate the position of the Bragg reflections. The asterisks indicate peaks belonging to impurities. For details on the results of the data treatment on all the powder patterns of Figure 6, the reader is referred to Tables 1 and 2 for benzene and cyclohexane, respectively. NP, IP1, IP2 = narrow-pore, intermediate-pore forms, respectively.

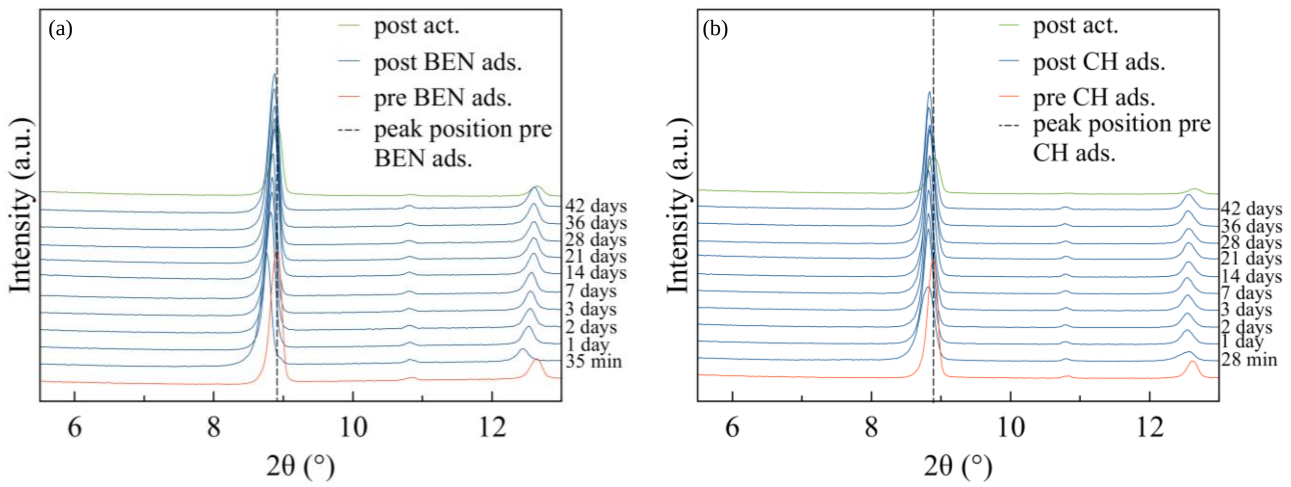

Figure S21. Powder X-ray diffraction patterns acquired as a function of time on the narrow-pore form of COF-300-rt recovered after impregnation with (a) benzene (BEN) and (b) cyclohexane (CH) (blue traces) at comparison with the pattern before impregnation (red traces) and that after impregnation and activation at 393 K and under vacuum for 12 hours (green traces). The fragmented line highlights the position of the [020] Bragg reflection pre-impregnation.

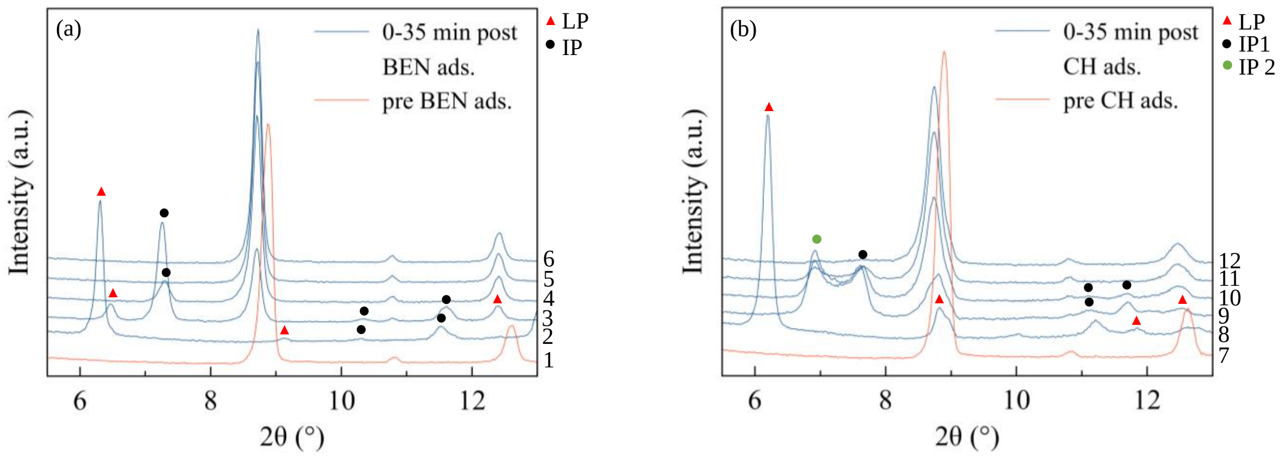

Figure S22. PXRD patterns of COF-300-rt acquired as a function of time, with steps of 7 minutes, before and after impregnation with (a) benzene (BEN) and (b) cyclohexane (CH). Immediately after benzene impregnation (0 min) the larger-pore and the intermediate-pore forms are present, the latter appearing during data acquisition (as evidenced by the absence of its [020] peak at *ca*. 7.2°). 14 minutes after the impregnation, only the intermediate-pore and the narrow-pore forms are detected, and 21 minutes after the impregnation the narrow-pore form is recovered alone. In the case of cyclohexane, the larger-pore form is detected with the narrow-pore form immediately after the impregnation (0 min). 7 minutes after the impregnation, the larger-pore form disappears, and two intermediate forms (IP1 and IP2) appear and persist, concomitant with the narrow-pore form, up to 21 minutes. Finally, 28 minutes after the impregnation, the narrow-pore form is recovered alone. NP, IP1, IP2 and LP = narrow-pore, intermediate-pore and larger-pore forms, respectively. 1, NP; 2, IP+LP; 3, NP+IP+LP; 4, NP+IP; 5, NP; 6, NP; 7, NP; 8, NP+LP; 9, NP+IP1+IP2; 10, NP+IP1+IP2; 11, NP+IP1+IP2; 12, NP.

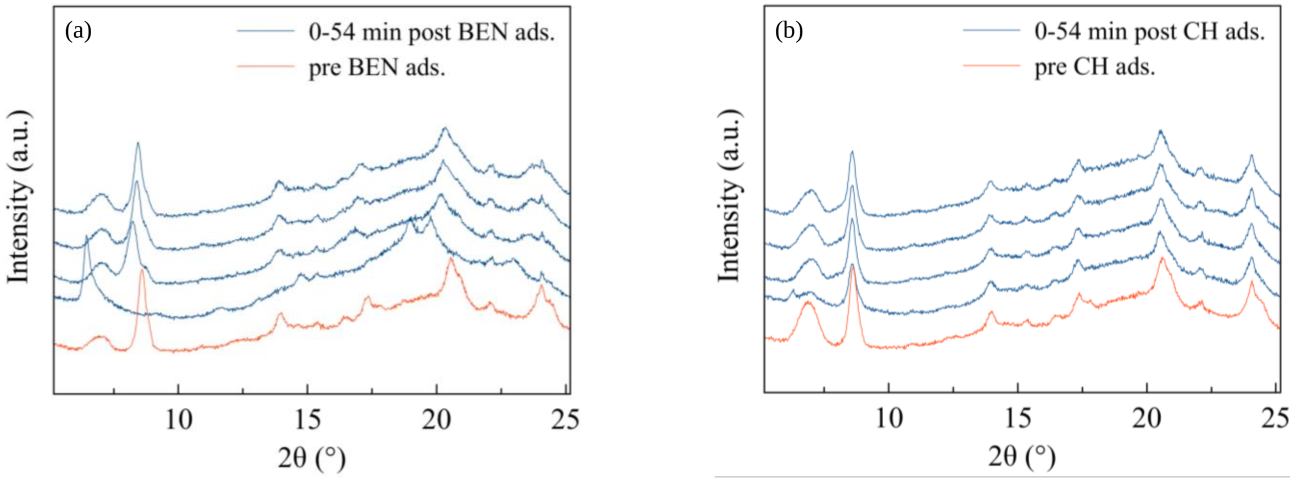

Figure S23. PXRD patterns of COF-300-st acquired as a function of time, with steps of 18 minutes, before and after impregnation with (a) benzene (BEN) and (b) cyclohexane (CH).

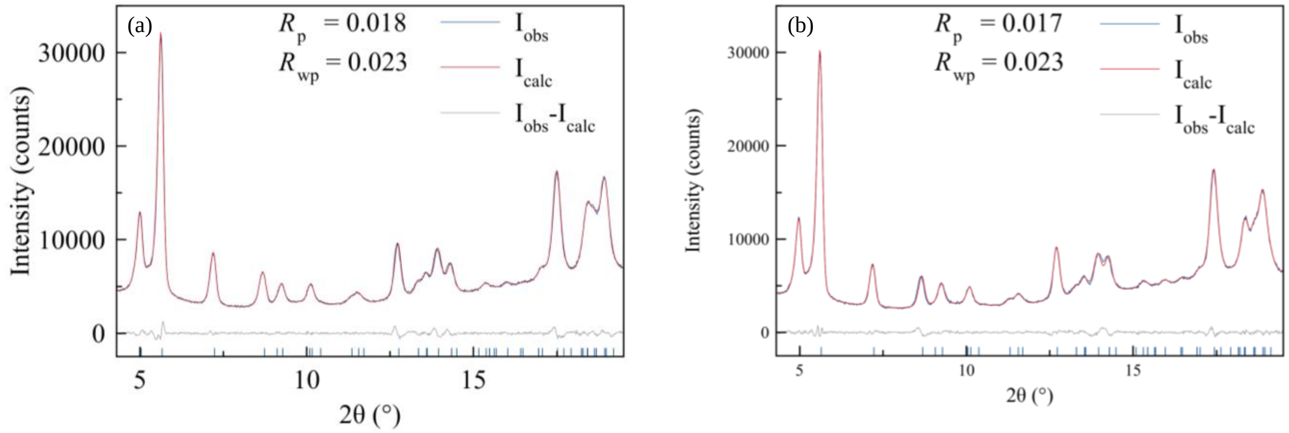

Figure S24. Graphical output (as a representative example) of the whole powder pattern refinement carried out on the data of LZU-111 acquired 13 minutes after the impregnation with (a) benzene and (b) cyclohexane. Observed, calculated and difference patterns: blue, red and grey traces, respectively. The ticks at the bottom indicate the position of the Bragg reflections. For details on the results of the data treatment on all the powder patterns of Figure 7, the reader is referred to Tables 3 and 4, for benzene and cyclohexane, respectively.

Section S3. Tables

Table S1. Information on the of the whole powder pattern refinements carried out for COF-300-rt after impregnation with benzene. NP, IP, LP = narrow-pore, intermediate-pore, larger-pore forms.

t (min)	Data	Parameters	R_p	R_{wp}	V (Å^3); ΔV/V$_{PRE}$ (%)		
					NP	IP	LP
Pre impr	1026	23	0.028	0.039	3472.2(4); 0	-	-
0	511[a]	16[a]	0.036[a]	0.048[a]	-	4352(4); 25.4[a]	5073(5); 46.1[a]
	456[b]	39[b]	0.022[b]	0.028[b]	3531(8); 1.7[b]	4480(4); 29.0[b]	5306(7); 52.8[b]
18	1026	31	0.028	0.037	3530.3(7); 1.7	4428(2); 27.5	-
36	1026	24	0.028	0.038	3532.7(6); 1.7	-	-
54	1026	21	0.029	0.039	3531.8(6); 1.7	-	-

[a] Values associated to the 2θ range 5.8-16.0° (see the Experimental Section); [b] values associated to the 2θ range 16.0-25.0° (see the Experimental Section).

Table S2. Information on the of the whole powder pattern refinements carried out for COF-300-rt after impregnation with cyclohexane. NP, IP1, IP2, LP = narrow-pore, intermediate-pore, larger-pore forms.

t (min)	Data	Parameters	R_p	R_{wp}	V (Å^3); ΔV/V$_{PRE}$ (%)			
					NP	IP1	IP2	LP
Pre impr	1026	28	0.033	0.043	3463.0(5); 0	-	-	-
0	456[a]	16[a]	0.022[a]	0.029[a]	3467(3); 1.8[a]	4078(4); 17.8[a]	4985(5); 44.0[a]	5373(8); 55.2[a]
	486[b]	20[b]	0.016[b]	0.020[b]	3545(2); 2.4[b]	4131(2); 19.3[b]	4930(2); 42.4[b]	-
18	1026	36	0.021	0.027	3520(1); 1.6	4081(3); 17.9	5129(4); 48.1	-
36	1026	31	0.023	0.029	3519.6(7); 1.6	-	-	-
54	1026	28	0.025	0.032	3523.0(7); 1.7	-	-	-

[a] Values associated to the 2θ range 5.5-16.2° (see the Experimental Section); [b] values associated to the 2θ range 16.2-25.5° (see the Experimental Section).

Table S3. Information on the of the whole powder pattern refinements carried out for LZU-111 before and after impregnation with benzene.

**t (min)**	**Data**	**Parameters**	$\boldsymbol{R}_{\mathbf{p}}$	$\boldsymbol{R}_{\mathbf{wp}}$	**V (Å^3)**	**ΔV/V$_{\mathbf{PRE}}$ (%)**
Pre impr	776	20	0.030	0.040	12304(3)	0
0	776	179	0.017	0.022	12025(2)	-2.5
13	776	179	0.018	0.023	12083(2)	-2.0
26	776	179	0.015	0.020	12135(3)	-1.5
39	776	179	0.014	0.018	12181(4)	-1.0
52	776	179	0.016	0.020	12221(5)	-0.6
65	776	179	0.017	0.022	12254(6)	-0.4
78	776	179	0.020	0.027	12281(7)	-0.1
91	776	179	0.022	0.030	12302(9)	0
104	776	179	0.024	0.031	12316(11)	0.1
117	776	179	0.024	0.032	12324(13)	0.1

Table S4. Information on the of the whole powder pattern refinements carried out for LZU-111 before and after impregnation with cyclohexane.

t (min)	Data	Parameters	R_p	R_{wp}	V (Å^3)	$\Delta V/V_{PRE}$ (%)
Pre impr	776	18	0.029	0.036	12295(3)	0
0	776	295	0.015	0.020	12139(2)	-1.2
13	776	295	0.017	0.023	12148(2)	-1.2
26	776	295	0.019	0.027	12158(2)	-1.3
39	776	295	0.019	0.028	12169(3)	-1.2
52	776	295	0.018	0.025	12180(4)	-1.1
65	776	295	0.018	0.026	12192(5)	-0.9
78	776	295	0.018	0.025	12205(6)	-0.8
91	776	295	0.019	0.025	12219(7)	-0.7
104	776	295	0.020	0.026	12234(8)	-0.5
117	776	295	0.021	0.027	12249(10)	-0.4
130	776	295	0.022	0.028	12266(11)	-0.3
143	776	295	0.023	0.029	12277(4)	-0.2
156	776	295	0.023	0.030	12286(2)	-0.1
169	776	295	0.023	0.030	12297(3)	0
182	776	295	0.024	0.032	12302(3)	0
195	776	295	0.024	0.032	12307(3)	0.1
208	776	295	0.025	0.034	12314(3)	0.1

Section S4. Calculation of the BET specific surface areas.

The following Table gathers details on the calculation of the Brunauer–Emmett–Teller (BET) specific surface areas of COF-300-rt, COF-300-st and LZU-111 retrieved from the N_2 adsorption isotherm at 77 K. The BET model to estimate the specific surface area was applied using the consistency criteria described by Rouquerol and co-workers.[5]

	**COF-300-rt**	**COF-300-st**	**LZU-111**
BET specific surface area (m^2/g)	39	1267	1834
Slope	0.107 ± 0.003	0.00343 ± 0.00006	0.00236 ± 0.00004
Y-intercept (g/cm^3 STP)	0.0037 ± 0.0004	0.000008 ± 0.000007	0.000003 ± 0.000003
C	30	453	747
Qm (g/cm^3 STP)	9	291	423
Correlation coefficient	0.999	0.999	0.998
Molecular cross-sectional area (nm^2)	0.1620	0.1620	0.1620
Number of picked points	7	11	7
Pressure range (p/p_0) for calculation	0.038–0.299	0.0049–0.2035	0.0050-0.1477

Section S5. References

(1) Ganesan, P.; Yang, X.; Loos, J.; Savenije, T. J.; Abellon, R. D.; Zuilhof, H.; Sudhölter. E. J. R. Tetrahedral *n*-type materials: efficient quenching of the excitation of *p*-type polymers in amorphous films. *J. Am. Chem. Soc.* **2005**, 127, 14530–14531. DOI: 10.1021/ja053689m.

(2) Plietzsch, O.; Schilling, C. I.; Tolev, M.; Nieger, M.; Richert, C.; Muller, T.; Bräse, S. Four-fold click reactions: Generation of tetrahedral methane- and adamantane-based building blocks for higher molecular assemblies. *Org. Biomol. Chem.* **2009**, 7, 4734–4743. DOI: 10.1039/B912189G.

(3) Pyka, I.; Ryvlin, D.; Waldvogel, S. R. Application of rigidity-controlled supramolecular affinity materials for the gravimetric detection of hazardous and illicit compounds. *ChemPlusChem* **2016**, 81, 926–929. DOI: 10.1002/cplu.201600296.

(4) SAIEUS Program, Version 3.0 by Jacek Jagiello, 2012-2019, NLDFT Models of Micromeritics Instrument Corp., 2000-2019.

(5) Rouquerol, J.; Llewellyn, P.; Rouquerol, F. Is the BET equation applicable to microporous adsorbents? In *Stud. Surf. Sci. Catal.*, Elsevier, Amsterdam and Oxford, **2007**; Vol. 160, pp. 49–56.
